# Impression management in distributive justice: balancing fairness and social perception

**DOI:** 10.3389/fpsyg.2026.1848060

**Published:** 2026-09-18

**Authors:** Gahyun Lim, Daeeun Kim, Hackjin Kim

**Affiliations:** 1 School of Psychology, Korea University, Seoul, Republic of Korea; 2 Department of Psychology, Korea Army Academy at Yeongcheon, Yeongcheon, Gyeongsangbuk-do, Republic of Korea

**Keywords:** competence, fairness, fear of negative evaluation, interoception, merit-based equity, warmth

## Abstract

Distributive justice involves choices between principles such as strict equality and merit-based equity. This study examined whether individuals use these principles strategically to manage impressions, signaling warmth or competence in resource allocation. In Experiment 1, allocation choices differed according to evaluator orientation, although evaluator selections were also linked to monetary incentives. In Experiment 2, after evaluator-linked monetary incentives were removed, a similar but attenuated descriptive pattern emerged; however, the critical interaction effects were not statistically significant. Thus, the findings suggest that social-evaluative orientation may influence allocation preferences, but this apparent influence was weaker without evaluator-linked monetary incentives and requires confirmation in more adequately powered studies. Furthermore, exploratory analyses tentatively suggested that individual differences in fear of negative evaluation and subjective interoceptive sensitivity might be preliminarily associated with variability in allocation choices across evaluator conditions, highlighting potential avenues for future targeted replication. These findings suggest that impression management influences fairness decisions, highlighting the social motivation underlying distributive preferences.

## 1 Introduction

As social beings, individuals often share resources acquired through cooperating or competing with others. The interdependent nature of outcomes necessitates social agreements on ‘fair’ resource allocation within and across groups, known as distributive justice (Deutsch, 1975; Cook and Hegtvedt, 1983). Two principal doctrines emerge within this concept: merit-based equity, endorsing distributing resources proportionally to individual contributions or efforts (Adams, 1965; Leventhal and Michaels, 1971; Cappelen et al., 2014), and strict equality, supporting an even distribution of resources, regardless of individual input (Deutsch, 1975; Goudarzi et al., 2022). These principles often conflict when group members have made unequal contributions, as evident in debates about real estate policies advocating higher taxes for multi-property owners, or in discussions about equitable financial aid distribution from government stimulus packages.

Individual preferences for these distributive justice principles can vary based on factors such as gender (Kahn et al., 1977; Caleo, 2018), age (Leventhal et al., 1972; Elenbaas, 2019), culture (Schäfer et al., 2015; Huppert et al., 2019), or individual characteristics such as risk aversion (Kameda et al., 2016) or compassion (Zhao et al., 2017). Moreover, an individual’s preference for a particular principle can dynamically shift based on their economic interests (DeScioli et al., 2014; Konow et al., 2020), as evident in people perceiving inequitable distribution as fair when it provides greater monetary incentives (DeScioli et al., 2014).

While previous research has demonstrated that monetary incentives can shape preferences for distributive principles (DeScioli et al., 2014; Konow et al., 2020), this study extends the inquiry by positing that individuals also adapt their fairness decisions based on anticipated social rewards. These rewards—such as being perceived as warm or competent—can act as powerful motivators in impression management (Leary and Kowalski, 1990), thereby influencing allocation behavior. For instance, political parties often solidify identities by aligning with arguments for either merit-based equity or strict equality on various issues (Graham et al., 2009; Haidt, 2012). Nevertheless, it remains unclear whether these social benefits influence individuals’ preferences for distributive justice principles, potentially as a form of impression management (Leary and Kowalski, 1990).

Therefore, this study examines whether preferences for merit-based equity versus strict equality can yield social rewards for decision-makers in the form of specific impressions, especially perceived warmth and competence. Furthermore, we explored whether individuals adjust their preferences as a form of impression management.

### 1.1 Impressions from strict equality vs. merit-based equity based on group goal

Previous research suggests strict equality and merit-based equity correspond to different group dynamics, with merit-based equity linked to competitive behaviors rewarding individual effort, while strict equality promotes cooperation through uniform distribution. These associations manifest in studies examining group norms, implying one’s behaviors following each principle reflect cooperative or competitive attitudes.

Firstly, merit-based equity was associated with increasing economic productivity, while strict equality was associated with maintaining harmony or preventing interpersonal conflicts (Deutsch, 1975; Shapiro, 1975; Konow et al., 2020). Participants viewed merit-based allocation as fairer when the group’s goal was productivity, while strict equality was perceived as fairer when the goal was group harmony (Kazemi et al., 2016). Group leaders often allocate resources based on merit or equally, depending on the group’s objectives (Leventhal et al., 1972; Kazemi et al., 2017).

Furthermore, these associations contribute to gender-based expectations in allocation, reinforcing stereotypes that suggest women are more cooperative and men are more competitive. Supporting this, females were more inclined to distribute resources equally, whereas males tended to allocate based on merit (Kahn et al., 1980). A female manager received fewer negative evaluations than a male manager when allocating equally in a group valuing individual performance (Caleo, 2018).

Despite this understanding, little research has explored the social rewards individuals might gain from choosing either strict equality or merit-based equity. Kahn et al. (1977) demonstrated varying evaluations of likability and power for allocators using these principles. However, this study employed a task where the allocator was also the recipient of the allocation, making it challenging to ascertain whether impressions formed based on the allocator’s actions or personal benefit sacrifice. To address this gap, the present study aims to investigate the social impressions of a third-party allocator whose performance or rewards are independent of their allocation decisions.

### 1.2 Inference of warmth and competence-based social impressions from individual behavior

When observing others’ actions, people form impressions broadly categorized as dimensions of socially good-bad and intellectually good-bad attributes (Rosenberg et al., 1968; Abele and Wojciszke, 2014). The “Stereotype Content Model” proposes these as warmth, referring to traits reflecting perceived intentions to benefit others, and competence, pertaining to traits reflecting perceived ability to execute those intentions (Fiske et al., 2002). These dimensions influence how individuals are perceived based on facial (Rudert et al., 2017), vocal (Ko et al., 2009) features, or reaction time (Evans and Rand, 2019; Jordan et al., 2016; Lim and Kim, 2022).

One’s warm or competent impression may signal underlying preferences for cooperative or competitive behaviors. Individuals can infer warmth or competence from cooperative resource allocation structures (Fiske and Bai, 2020), expecting less warmth in competitive relationships (Russell and Fiske, 2008; Fiske, 2015, 2018) but predicting competence based on social inequality (Oldmeadow and Fiske, 2010; Durante et al., 2013; Durante and Fiske, 2017).

Moreover, other’s warm or competent impressions could influence how they interpret resource distribution or react, such as judgments of morality (Cikara et al., 2010; Lee et al., 2014; Rom et al., 2017; Rudert et al., 2017; Azevedo et al., 2018) or fairness (Lim and Kim, 2022). For example, dictators in the dictator game were likely to avoid receiving more than warm recipients but less than competent recipients (Jenkins et al., 2018; Kobayashi et al., 2022).

Given that strictly equal or merit-based allocation often guides cooperative or competitive interactions, individuals may infer different warmth and competence based on others’ preferences. Conversely, people may express distributive justice preferences as impression management.

### 1.3 Impression management behaviors

Impression management involves psychological and cognitive manipulation of how one’s self is perceived (Leary and Kowalski, 1990), evident in various social behaviors. When observed, individuals tend to exhibit more socially desirable behaviors like prosocial behaviors (Izuma et al., 2010; Izuma, 2012; Jung et al., 2018), punishing non-cooperators (Kurzban et al., 2007; Peterburs et al., 2017), or social conformity (Cialdini and Goldstein, 2004; Wei et al., 2013; Kim et al., 2021). Alternatively, depending on contextual or personal preferences, people may manage only specific impressions (Bolino and Turnley, 2003; Weiss and Feldman, 2006; Holoien and Fiske, 2013; Yoon et al., 2021), using self-promotion to appear more competent or ingratiation to seem more amiable (Stevens and Kristof, 1995; Turnley and Bolino, 2001). They may also conceal undesirable traits (Curtis et al., 2023) and feign desirable traits for job requirements (Lindholm and Yzerbyt, 2018).

What motivates impression management? Recent studies suggest that the brain engages in allostatic prediction to maintain physiological homeostasis, motivating the minimization of prediction errors that could entail metabolic costs (Barrett and Simmons, 2015; Pezzulo et al., 2015). This mechanism extends to social contexts, including inference and interaction, where people construct internal models of others’ minds to predict behaviors (FeldmanHall and Shenhav, 2019; Veissière et al., 2020) by abstracting stable traits from temporary behaviors (Evans and van de Calseyde, 2018; Jordan et al., 2016; Lim and Kim, 2022; Thornton et al., 2019) or leveraging known traits to predict imminent behavior (Becker and Asbrock, 2012; Evans and Krueger, 2016; Bach and Schenke, 2017; Simon et al., 2020; Tamir and Thornton, 2018). Following this process of social inference, individuals might adapt behaviors to align with inferred expectations (Constant et al., 2019; Theriault et al., 2021), avoiding future uncertainty.

People often tailor their impression management strategies to suit specific contextual demands or personal objectives (Bolino and Turnley, 2003; Weiss and Feldman, 2006; Holoien and Fiske, 2013; Yoon et al., 2021). Furthermore, they may selectively emphasize traits such as warmth or competence to project a particular image (Swencionis and Fiske, 2016). These adaptive behaviors reflect an individual’s ability to gauge observers’ expectations and adjust their actions accordingly. In social interactions, people construct internal models of others’ minds to predict behaviors (Tamir and Thornton, 2018; Veissière et al., 2020) by inferring social traits or forecasting future actions from current behaviors (Jordan et al., 2016; Lim and Kim, 2022; Thornton et al., 2019).

### 1.4 Overview of this study

This study investigates whether individuals modify their preference for strict equality versus merit-based equity as a means of impression management in social interactions. Specifically, we explore whether individuals strategically alter their distribution decisions to signal either warmth or competence in the presence of evaluators who explicitly value these traits. This process aligns with impression management theories suggesting that individuals actively shape their social image through behavioral adjustments (Leary and Kowalski, 1990). To empirically investigate this impression management process, we conducted two online experiments in a virtual laboratory setting.

In Experiment 1, monetary incentives were introduced to stimulate impression management motives. In Experiment 2, monetary incentives were removed to isolate the influence of social rewards. In other words, to test the motivational basis of fairness-based impression management, we compared conditions where impression gains could be linked to tangible rewards (monetary incentives) versus conditions where rewards were purely social (e.g., favorable evaluation). We hypothesized that the presence of monetary incentives would enhance the salience of impression motives, as participants had a financial reason to adjust their behavior. Conversely, in the absence of monetary rewards, any behavioral shift would more likely reflect the value of social evaluation alone. This complementary design allowed us to examine a baseline paradigm in Experiment 1 where impression-related and strategic monetary motives were instrumentally intertwined, and subsequently contrast it with Experiment 2, where direct monetary incentives were entirely eliminated to isolate social evaluative concerns.

Participants performed a task in which they acted as third-party allocators, distributing points between two competing recipients. Their own monetary gains were unaffected by their decisions, ensuring an impartial role. For each trial, participants chose between equal distribution and merit-based allocation of points, knowing that their decisions would be observed by one of two evaluators—one valuing warmth and the other competence.

Additionally, we accounted for the impact of efficiency in resource distribution. Previous research suggests that fairness and efficiency are both significant factors in decision-making (Hsu et al., 2008; Gordon-Hecker et al., 2017). For example, efficiency is often prioritized over fairness when resources are scarce (Chae et al., 2022). To examine this, we adjusted point distributions across trials to represent varying levels of efficiency.

Our study had three primary objectives. First, we examined whether an evaluator valuing warmth would encourage a preference for strict equality, while one favoring competence would promote merit-based equity. Second, we sought to determine whether participants adapted their distribution preferences to align with the traits valued by the evaluator. Finally, we measured individual differences in impression management by calculating the variability of preferences across different evaluator conditions. We also explored the relationships between this variability and related survey measures.

## 2 Experiment 1

In Experiment 1, we explored whether individuals, acting as third-party allocators, change how they distribute resources to influence others’ perceptions of them, particularly in terms of warmth and competence. Specifically, we examined whether individuals shift their preferences between equal distribution (e.g., winner: loser = 5:5) and merit-based distribution (e.g., winner: loser = 6:4) depending on whether they are being observed by an evaluator favoring either a warm or competent impression. Moreover, we investigated whether equal distribution is more often linked to warmth and merit-based distribution to competence.

### 2.1 Methods

#### 2.1.1 Participants

Sixty-nine participants (41 women, mean age = 23.17 years, age range = 19–35) were recruited from online communities of several universities located in Seoul, South Korea. The sample size for Experiment 1 was determined by an *a priori* power analysis using G*Power 3.1.9.7 software. The effect size for the sample size (interaction effect: partial *η*^2^ = 0.05) was drawn from a previous study (Rom and Conway, 2018) that measured impression management for warmth and competence in a moral dilemma, resulting in a required sample size of 43 with 80% power in a repeated-measures MANOVA with a 5% false positive rate. In the data analyses, we excluded eight participants: one for data loss, one for suspicion of other participants’ presence, and six for experimental manipulation failure (one did not perceive the warm evaluator as warm, and five did not perceive the competent evaluator as competent). Therefore, 61 participants (38 women, mean age = 22.97 years, age range = 19–35) were included in the data analyses. When we conducted a sensitivity power analysis using G*Power, our final sample size of 61 would be able to detect a minimum effect size of *f* = 0.15 for interaction effects, with an 80% power and a 5% false positive rate. All included participants were healthy without any history of mental disorders. Participants provided informed consent, and the protocol was approved by the Institutional Review Board of Korea University. Participants received KRW 15,000–21,000 (approximately USD 11–16) as compensation, including KRW 15,000 for the 90-min behavioral experiments and additional monetary incentives up to KRW 6,000 for the main task, memory task, and surveys.

#### 2.1.2 Task design and procedure

##### 2.1.2.1 Experimental procedure

In our experiments, participants interacted with others through avatars in a virtual setting. The experimenter explained that the study was designed to measure participants’ cognitive abilities and social behaviors in a metaverse environment. Each experiment involved four individuals: two real participants and two confederates. These individuals were assigned roles as either “allocators” or “evaluators.” While participants were informed that roles were assigned randomly, real participants were always assigned the role of allocators.

The experiment consisted of two phases: a ‘cognitive task’ and a ‘social allocation task (the main task)’ (Figure 1A). Each phase included tasks specific to the participants’ roles. During the cognitive task, the allocators engaged in a memory task, while the evaluators performed a dot estimation task. The cognitive task was designed to manipulate the evaluator condition, by associating each evaluator with a valued social impression—either warmth or competence. Allocators had to memorize the correct pairings between evaluators and their valued social traits.

**Figure 1:**
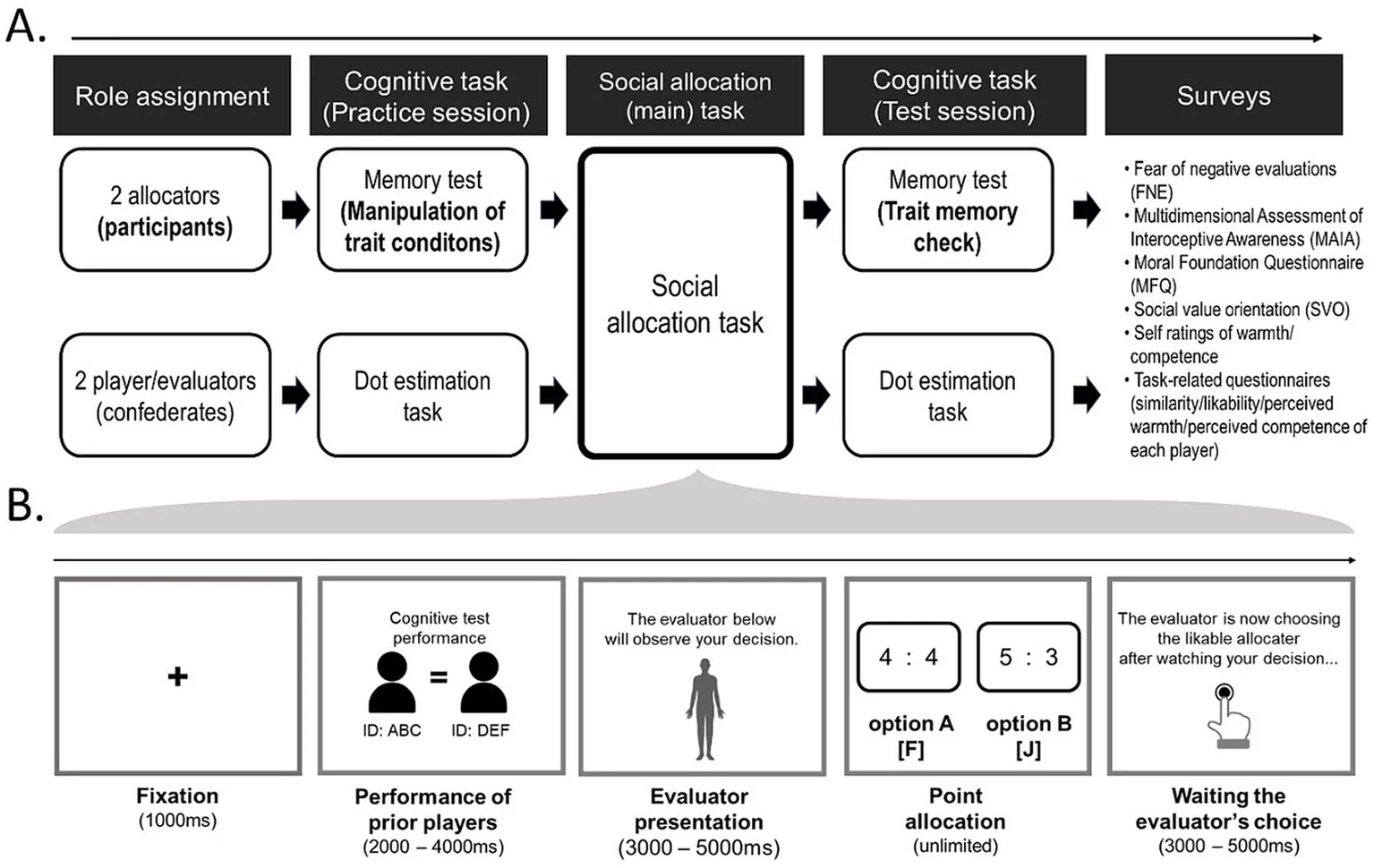
The experimental procedure and task screens of the social allocation task. **(A)** Upon visiting the online laboratory, two real participants were assigned as allocators, while two confederates were assigned as evaluators. Participants then practiced cognitive tasks, which varied based on their role (the dot estimation task for evaluators and the memory task for allocators). In the memory task, allocators memorized the impression traits valued by each evaluator (e.g., four warmth-related traits or four competence-related traits). Next, all participants performed the social allocation task, where allocators distributed points to the previous participants while being observed by one of two evaluators, each valuing either warmth or competence. After the social allocation task, all participants carried out the test session of the cognitive task. Finally, participants completed surveys and were debriefed. **(B)** In each trial, participants could see whether the prior participants had tied or if one had won the dot estimation task. Then, the evaluator for the trial was randomly selected and displayed on the screen. The two allocators decided how to distribute the prior participants’ points between equal and unequal point distributions. Afterward, they waited for the evaluator to choose their preferred allocator based on the observed point distributions.

Following the cognitive task, all participants moved to the social allocation task. In each trial (Figure 1B), allocators viewed results of a dot estimation task completed by two anonymous participants in prior experiment sessions. They then allocated points to these prior participants, choosing either an equal distribution (Option A) or an unequal distribution (Option B). Allocators were told one evaluator, known to prioritize either warmth or competence, would observe the allocations from two allocators and select their preferred option. Because the evaluator’s selection in Experiment 1 was explicitly linked to monetary incentives, participants’ allocation choices potentially reflect a conflation of both impression-management concerns and strategic instrumental motives aimed at maximizing the probability of being rewarded. To ensure allocators treated each trial independently and avoided calculating cumulative points, the task design involved allocating points to different anonymous participants across multiple trials.

Following the social allocation task, allocators performed the memory task (“test session”) to assess whether allocators retained their understanding of the evaluators’ valued social impressions. Finally, participants completed surveys and were debriefed. Further procedural and task details can be found in sections 2.1.2.2–2.1.2.4 and Supplementary material.

##### 2.1.2.2 Cognitive task

Participants were told that their cognitive abilities would be evaluated through different tasks based on their roles. Allocators (actual participants) undertook a memory task, while evaluators (confederates) completed a dot estimation task. Both tasks were executed twice—before and after the social allocation task—referred to as the practice and test sessions, respectively. We told participants that the test session would be a crucial assessment of their cognitive ability; however, it was actually designed to shape the evaluators’ impressions during the practice session prior to the social allocation task.

In the memory task, allocators had to remember four traits that each of two evaluators most valued, which were submitted during the pre-visit phase. The task displayed two character images representing each evaluator, each shown alongside four traits they valued most. The ‘warm’ character image was paired with warmth-related traits (‘warm’, ‘friendly’, ‘well-intentioned’, and ‘good-natured’). The ‘competent’ character image was paired with competence-related traits (‘competent’, ‘intelligent’, ‘confident’, and ‘skillful’). To minimize demand characteristics, these evaluators were not explicitly labeled as ‘warm evaluator’ or ‘competent evaluator’.

Each trial presented one evaluator’s character image with up to four trait words. Allocators judged whether all of the suggested traits correctly matched the character image. In the practice session, participants were required to achieve at least 90% accuracy; otherwise, they repeated the entire set of trials. Following the social allocation task, participants completed the test session to assess whether the associations were retained. Further details of the cognitive task are provided in Supplementary material (Text 1.3).

##### 2.1.2.3 Social allocation task (main task)

In the social allocation task, each participant, as an allocator, chose between two options for distributing points to two recipients who had participated in previous experimental sessions. Current evaluators observed both allocators’ choices and chose their preferred allocator. To motivate allocators for impression management, a competitive framework was designed, with allocators earning financial incentives—up to KRW 5,000—based on their preference rates by current evaluators.

Each trial began by displaying the dot estimation performance of recipients, represented by ‘=’ (‘Tie’ condition) or ‘≠’ (‘Win’ condition) symbols. The ‘=’ symbol indicated a tie between recipients, while ‘≠’ indicated the presence of a winner. The identities of recipients (e.g., initials) were changed in every trial to prevent participants from tracking cumulative points. Subsequently, a screen displayed an image indicating the evaluator of the current trial. Three types of images could be presented: the character valuing warmth-related traits, the character valuing competence-related traits, and a mosaic (control condition where allocators were not informed of which evaluator would observe them).

Two options for point allocation appeared in rounded squares: Option A (left) for equal allocation and Option B (right) for unequal allocation. Option A always gave equal points to both recipients. In the ‘Win’ condition, Option B gave more points to the winner; in the ‘Tie’ condition, it randomly favored one recipient. After choosing, participants awaited the evaluator’s decision on which allocator they preferred.

The study aimed to explore the conflict between merit-based (equitable) and strictly equal distribution. In a ‘Tie’ condition, Option A was equal and merit-based, while option B was unequal and non-merit-based. Conversely, in a ‘Win’ condition, Option A was equal but non-merit-based, whereas Option B was merit-based but unequal. Analyses primarily focused on the ‘Win’ condition, where equality and merit-based equity conflicted. The inclusion of the “Tie” condition is intended to serve as a neutral comparison point, in which merit-based allocation is not clearly justified by superior performance. This allows us to assess whether participants shift their distributive preferences primarily when performance-based justification is salient (‘Win’ condition), or whether impression management also operates under ambiguous merit conditions (‘Tie’ condition).

We also considered the efficiency of each option, calculated as the sum of points allocated. The difference in points between unequal and equal options was defined as ΔEfficiency, with levels ranging from −4 to 4 at 2-point intervals. Equal options had three variations of even allocations (4:4, 5:5, or 6:6) to prevent predetermined decisions. Unequal options had fixed differences of two points (e.g., 3:5, 4:6). For each equal option, unequal options were defined with five levels of ΔEfficiency (e.g., 1:3, 2:4, 3:5, 4:6, and 5:7 for the 4:4 equal option). These 15 combinations were uniformly suggested across each evaluator condition, presented once for the ‘Tie’ condition and twice for the ‘Win’ condition. Each participant encountered 135 trials, requiring 35–40 min for the social allocation task.

##### 2.1.2.4 Surveys and post-experiment questionnaires

At the end of the experiment, participants completed surveys related to preferences for distributive justice principles, including the Moral Foundation Questionnaire (MFQ; Graham et al., 2009) and Social Value Orientation (SVO; Murphy et al., 2011). Additional surveys examined sensitivity to social rewards and bodily signals, including the Brief Fear of Negative Evaluation Scale (BFNE-2; Carleton et al., 2007) and Multidimensional Assessment of Interoceptive Awareness (MAIA-2; Mehling et al., 2018). Participants also self-rated their warmth and competence (Fiske et al., 2002), and evaluated the characters of two evaluators in terms of similarity, likability, and perceived warmth and competence on a four-point Likert scale (1 = very unlikely, 4 = very likely). Six participants were excluded for rating low (<2) warmth for the warm-valuing evaluator or low (<2) competence for the competence-valuing evaluator, indicating ineffective trait manipulation. An additional participant was excluded during debriefing for doubting the authenticity of other participants.

#### 2.1.3 Statistical analyses

We conducted a 2 (Performance: Tie or Win) × 3 (Evaluator types: Warmth-valuing, Control or Competence-valuing) × 5 (ΔEfficiency levels: −4, −2, 0, 2, or 4) repeated-measures ANOVA (RM-ANOVA) on the proportion of the unequal allocation choice or reaction time (RT). Choices and RT data were averaged across three levels of equal allocation (i.e., 4:4, 5:5, or 6:6) and RT data were normalized to z-scores. Outliers in RTs, defined as RTs exceeding three standard deviations from individual means, were excluded from the analyses. To explicitly control for Type I error rate across multiple comparisons, Bonferroni corrections were strictly applied to all post-hoc pairwise comparisons and simple main effect analyses. All analyses were performed using SPSS 26.0. Effect sizes with 95% confidence interval (CI) were calculated using the R package effectsize v1.0.0, employing the ‘F_to_eta2’ function for partial *η*^2^ in RM-ANOVA and the ‘t_to_d’ function for Cohen’s dz. in paired *t*-tests.

### 2.2 Results

#### 2.2.1 Choice data

This experiment investigated whether an allocator’s distribution decisions are influenced by the recipients’ performance, the efficiency of distributive options, and the social impression valued by the evaluator. We conducted a 2 (Performance: Tie or Win) x 3 (Evaluator Types: Warmth-valuing, Control, Competence-valuing) × 5 (ΔEfficiency levels: −4, −2, 0, 2, 4) RM-ANOVA on the proportion of unequal allocation choices.

There was a significant three-way interaction of evaluator types, performance, and ΔEfficiency levels (*F*(8, 53) = 2.57, *p* = 0.019, partial *η*^2^ = 0.28, 95% CI = [0.01, 0.40]; Figure 2). Parsing this interaction revealed that the interaction between evaluator types and ΔEfficiency levels was significant only in the ‘Win’ condition (‘Win’ condition: *F*(8, 53) = 3.43, *p* = 0.003, partial *η*^2^ = 0.34, 95% CI = [0.06, 0.47]). In the ‘Win’ condition, allocators were more likely to favor strictly equal allocations when observed by warmth-valuing evaluators compared to the control condition, regardless of ΔEfficiency levels. On the other hand, they favored merit-based allocations when observed by competence-valuing evaluators over the control condition, but only when the unequal allocation was inefficient.

**Figure 2:**
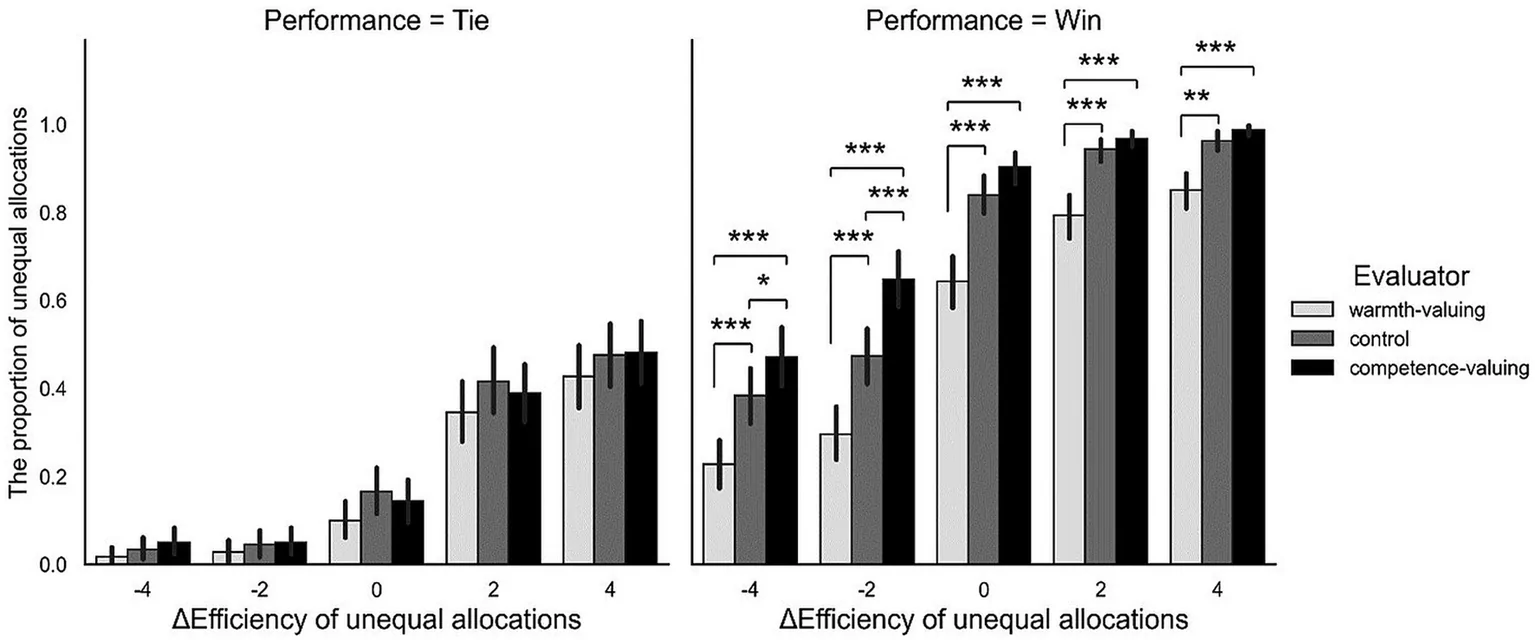
Bar plots of the choice rates representing the Performance × Evaluator × ΔEfficiency interaction effect. In Experiment 1, the three-way interaction between performance, evaluator, and ΔEfficiency was significant in the choice rate of unequal allocations. Notably, in the ‘Win’ condition, where strict equality and merit-based equity conflicted, the type of evaluator significantly influenced preferences between strict equality and merit-based equity. This influence was particularly pronounced when strict equality yielded greater efficiency than merit-based equity. Error bars indicate the 95% confidence interval. The asterisk indicates the significance level as follows: * (*p* < 0.05), ** (*p* < 0.01), and *** (*p* < 0.001).

Additionally, we found a significant evaluator type × performance interaction effect (*F*(2, 59) = 15.53, *p* < 0.001, partial *η*^2^ = 0.34, 95% CI = [0.15, 0.50]) and a main effect of the evaluator type (*F*(2, 59) = 25.84, *p* < 0.001, partial *η*^2^ = 0.47, 95% CI = [0.27, 0.60]). Allocators generally preferred equal allocations under warmth-valuing evaluators (Mwarmth-valuing ± SEM = 0.37 ± 0.02, Mcontrol ± SEM = 0.47 ± 0.01; control > warmth-valuing: *t*(60) = 6.31, *p* < 0.001, Cohen’s dz. = 0.81, 95% CI = [0.52, 1.10]) and unequal allocations under competence-valuing evaluators compared to control conditions (Mcompetence-valuing ± SEM = 0.51 ± 0.01; competence-valuing > control: *t*(60) = 3.89, *p* < 0.01, Cohen’s dz. = 0.50, 95% CI = [0.23, 0.77]). This tendency was stronger in the ‘Win’ condition, where the equal option indicated strict equality and the unequal option indicated merit-based equity.

There were also significant main effects of performance (*F*(1, 60) = 148.44, *p* < 0.001, partial *η*^2^ = 0.71, 95% CI = [0.59, 0.79]) and ΔEfficiency (*F*(4, 57) = 28.35, *p* < 0.001, partial *η*^2^ = 0.67, 95% CI = [0.51, 0.76]), showing that allocators preferred to allocate more points to the winners and that they preferred the option with a higher total distribution. The interaction between performance and efficiency was also significant (*F*(4, 57) = 13.97, *p* < 0.001, partial *η*^2^ = 0.49, 95% CI = [0.29, 0.62]), suggesting that allocators were more likely to choose unequal distributions as they became more efficient in the ‘Win’ condition. The evaluator × ΔEfficiency interaction was not statistically significant (*F*(8, 53) = 1.62, *p* = 0.14, partial *η*^2^ = 0.20). Full statistical results of choice data are provided in Supplementary Tables S1, S2.

#### 2.2.2 RT data

Previous studies suggest that RT can serve as an implicit measure of cognitive effort and decision conflict (Evans and Rand, 2019). If participants experience greater cognitive conflict when attempting to balance fairness and impression management, we would expect longer RTs when allocators face warmth-valuing evaluators compared to competence-valuing evaluators. This aligns with the idea that decisions involving social trade-offs often require more deliberation (FeldmanHall and Shenhav, 2019). Therefore, we examined the effects of recipients’ performance, evaluator type, and the efficiency of options on individually standardized reaction time (RT) data.

The 2 (Performance) × 3 (Evaluator Type) × 5 (ΔEfficiency Level) RM-ANOVA revealed significant performance × evaluator type interaction (*F*(2, 59) = 9.47, *p* < 0.001, partial *η*^2^ = 0.24). Bonferroni-corrected post-hoc analyses showed that, in the ‘Win’ condition, RTs were significantly slower under the warmth-valuing evaluator (vs. Control: *t*(60) = 3.25, *p* = 0.006, Cohen’s dz. = 0.42, 95% CI = [0.15, 0.68]) and significantly faster under the competence-valuing evaluator (vs. Control: *t*(60) = −2.67, *p* = 0.031, Cohen’s dz. = −0.34, 95% CI = [−0.60, −0.08]), both relative to the control condition. However, in the ‘Tie’ condition, there were no significant differences between evaluator conditions (Warmth-valuing vs. Control: *t*(60) = −1.02, *p* = 0.916; Competence-valuing vs. Control: *t*(60) = −0.70, *p* = 1.000). These results might suggest that participants may have considered both social impression management and equity concerns; RTs increased when these motives conflicted, and decreased when they were aligned, relative to the control condition.

Additionally, a significant interaction effect of evaluator type × ΔEfficiency level was found (*F*(8, 53) = 3.37, *p* = 0.003, partial *η*^2^ = 0.34, 95% CI = [0.06, 0.47]). RTs were longer under the warmth-valuing than the competence-valuing evaluator when unequal allocations were more efficient.

The performance × ΔEfficiency level interaction was also significant (*F*(4, 57) = 10.24, *p* < 0.001, partial *η*^2^ = 0.42, 95% CI = [0.20, 0.56]). Participants responded more quickly in the ‘Tie’ condition and more slowly in the ‘Win’ condition when the efficiency of the unequal option was low. This would reflect the task structure, which encourages choosing equal options in the ‘Tie’ condition and unequal options in the ‘Win’ condition.

Main effects were significant for performance (*F*(1, 60) = 30.89, *p* < 0.001, partial *η*^2^ = 0.34, 95% CI = [0.16, 0.50]) and evaluator type (*F*(2, 59) = 5.74, *p* = 0.005, partial *η*^2^ = 0.16, 95% CI = [0.02, 0.32]). No other significant effects were observed (ΔEfficiency: *F*(4, 57) = 1.82, *p* = 0.14, partial *η*^2^ = 0.11; evaluator type × performance × ΔEfficiency interaction: *F*(8, 53) = 0.98, *p* = 0.46, partial *η*^2^ = 0.13). Full statistical results of RT data are provided in Supplementary Tables S3, S4.

#### 2.2.3 Individual differences in the extent of impression management

To explore potential correlations between individual differences in impression management and personal traits, we calculated the L2-norm distances between allocation choices made under the conditions of warmth-valuing or competence-valuing evaluators, compared to a control condition. Specifically, for each participant, we represented their choice rates across all efficiency levels and performance conditions as vectors, and computed the Euclidean distance (L2-norm) between each evaluator vector and the control vector. The resulting value indicates the extent to which an individual’s choice pattern deviates from their baseline (control) behavior, thus reflecting behavioral adjustment due to impression management.

For personal traits, we focused on surveys related to preferences on moral principles, sensitivity to social evaluation, and interoceptive sensitivity. Subjective interoceptive sensitivity, as measured by the MAIA-2 score, negatively correlated with the degree of impression management when observed by a competence-valuing evaluator vs. control condition [*r*(58) = −0.27, *p* = 0.038]. However, subsequent analysis revealed that the correlation was no longer significant after excluding an outlier beyond 3 SD [*r*(57) = −0.16, *p* = 0.231]. Full correlation results are provided in Supplementary Table S5.

### 2.3 Discussion

This experiment demonstrated that participants adjusted their preferences between two principles of distributive justice—strict equality and merit-based equity—in response to the impressions valued by evaluators. Specifically, a preference for strict equality emerged when evaluators valued warmth, whereas a preference for merit-based equity was apparent when competence was valued. These findings hold significant implications. First, they suggest that warm impressions align with strict equality, while competent impressions correlate with merit-based equity. Second, they indicate that decisions regarding fairness can be influenced not only by inherent values but also by the social impressions individuals seek to convey.

Furthermore, the efficiency of the available options moderated the impact of evaluator type on participants’ preferences for strict equality vs. merit-based equity. Notably, when the strictly equal allocation was more efficient, the influence of evaluators became more pronounced. This phenomenon might be attributed to a potential ceiling effect, as the impact of the competence-valuing evaluator appeared constrained, possibly due to a general bias towards merit-based allocation when recipients’ performances varied.

Our experimental design allowed participants to simultaneously acquire both social impressions and monetary incentives when making their choices. Consequently, there is a possibility that financial incentives, rather than impression management, might drive their decisions. Additionally, the monetary incentive might have been too strong, potentially obscuring individual differences in impression manipulation. Therefore, we conducted Experiment 2, where we removed monetary rewards to better understand the role of social motivations in shaping preferences for strict equality versus merit-based equity.

## 3 Experiment 2

### 3.1 Methods

#### 3.1.1 Participants

Forty-one participants (29 women, mean age = 23.85 years, age range = 19–35) were recruited from several universities located in Seoul, South Korea, through online communities. The sample size for Experiment 2 was determined based on the three-way interaction effect (partial *η*^2^ = 0.28) observed in Experiment 1. An *a priori* power analysis suggested a sample size of 32 participants, providing 80% power in a repeated-measures MANOVA with a 5% false positive rate. Access to the online laboratory was provided via URL links in their web browsers. Seven participants were excluded from the data analysis: three due to suspicion about the presence of other participants, and four for failing to correctly manipulate the evaluators’ types. Thus, data analyses were conducted on the remaining 34 participants (28 women, mean age = 23.38 years, age range = 19–35). When we conducted a sensitivity power analysis using G*Power, our final sample size of 34 would be able to detect a minimum effect size of *f* = 0.40 for interaction effects, with an 80% power and a 5% false positive rate. All participants included in the data analyses were healthy and had no history of mental disorders. They provided informed consent before participating, and the experimental protocol was approved by the Institutional Review Board of Korea University. Participants received a participation fee of KRW 17,000–20,000 (approximately USD 14–16), which includes KRW 17,000 (100 min; KRW 10,000 per hour) for the behavioral experiments and additional monetary incentives of up to KRW 3,000 for completing memory tasks (maximum KRW 2,000) and surveys (maximum KRW 1,000).

#### 3.1.2 Task design and procedure

Experiment 2 followed the procedures and task structures of Experiment 1 but introduced specific changes to eliminate monetary incentives. Participants were informed they would engage in three tasks, instead of two, and were also instructed to turn on their cameras and microphones for a “cooperative task” which was to be the final task. However, this final task was never implemented and was designed to subtly suggest that evaluators’ preferences might influence it. No monetary incentives were linked with the evaluators’ choices. Instead, at the end of a memory test, participants were shown the proportion of trials where each allocator was preferred, presented as potentially impactful for the non-existent final task.

We adopted the Korean Multidimensional Assessment of Interoceptive Awareness (K-MAIA; Gim et al., 2016) to measure interoceptive sensitivity, replacing the original MAIA-2 scales. We employed the K-MAIA in Experiment 2 because it is a validated Korean adaptation of the MAIA developed for Korean samples (Gim et al., 2016). In the K-MAIA validation study, the scale was reconstructed after item analysis, including consideration of possible method effects associated with positively and negatively worded items. Accordingly, we selected the validated Korean version for use in Experiment 2. Minor adjustments were made: the ‘≠’ sign was replaced with the ‘>’ sign to indicate the ‘Win’ condition, and the memory test reward per correct answer was increased from KRW 50 to KRW 150, compensating for the absence of evaluator-based incentives.

#### 3.1.3 Statistical analyses

We performed a three-way RM-ANOVA with factors: 2 performance levels (Tie or Win), 3 evaluator types (warmth-valuing, competence-valuing, control), and 5 ΔEfficiency levels (−4, −2, 0, +2, +4). The analysis was applied to the proportion of unequal allocation choices and RT. We averaged data at equal allocation levels (i.e., 4:4, 5:5, and 6:6) and normalized RT data to z-scores. In RT data analyses, trials with RTs deviating by more than 3 SD from individual means were excluded. To explicitly control for Type I error rate across multiple comparisons, Bonferroni corrections were strictly applied to all post-hoc pairwise comparisons and simple main effect analyses. All analyses were performed using SPSS 26.0. Effect sizes with 95% CI were calculated using the R package effectsize v1.0.0, employing the ‘F_to_eta2’ function for partial *η*^2^ in RM-ANOVA and the ‘t_to_d’ function for Cohen’s dz. in paired *t*-tests.

Furthermore, we conducted exploratory analyses to investigate the association between individual differences in impression management and moral preferences, social evaluation sensitivity, and interoceptive sensitivity. The degree of a participant’s impression management behavior was defined as the extent to which participants modified their allocations depending on the evaluator’s valuing impressions. This behavioral change was quantified using L2-norm distances from the control condition choices for each evaluator type. These analyses focused on the ‘Win’ condition where strict equality and merit-based equity conflicted.

For individual characteristics, we used sub-scores from the Moral Foundations Questionnaire (MFQ) and SVO measures to gauge moral preferences. The individual motivation to avoid negative impressions was quantified using BFNE-2 scores, while the capacity for interoceptive signal detection and regulation was assessed via K-MAIA sum scores. Pearson correlations were calculated between these individual metrics and the degree of behavioral modification. Additionally, we applied these analyses to data from Experiment 1 to discern whether these individual characteristics could explain variance in impression management behavior more generally, or specifically when monetary motivation was absent.

For both studies, we report all measures, manipulations, and data exclusions. All relevant information is included in the manuscript and the Supplementary material. Behavioral data and codes are publicly available on the Open Science Framework (OSF) at https://osf.io/xu9n6/.

### 3.2 Results

#### 3.2.1 Choice data

In Experiment 2, we conducted a 2 (Performance) × 3 (Evaluator Type) × 5 (ΔEfficiency Level) RM-ANOVA to examine the influence of the evaluator types on allocation choices without monetary incentives. Overall, the influence of evaluator type was reduced compared to Experiment 1.

The three-way interaction effect [*F*(2, 32) = 0.35, *p* = 0.70, partial *η*^2^ = 0.02] and the evaluator type × performance interaction [*F*(2, 32) = 1.20, *p* = 0.31, partial *η*^2^ = 0.07] were not statistically significant. On the other hand, the main effect of evaluator type remained significant [*F*(2, 32) = 3.74, *p* = 0.035, partial *η*^2^ = 0.19; Figure 3A]. Bonferroni-corrected post-hoc analyses showed that participants were more likely to choose unequal allocations under the competence-valuing evaluator than the warmth-valuing evaluator (Competence-valuing vs. Warmth-valuing: *t*(33) = 2.68, *p* = 0.027, Cohen’s dz. = 0.47, 95% CI = [0.10, 0.82]), although neither differed significantly from the control condition (Warmth-valuing vs. Control: *t*(33) = −2.46, *p* = 0.072, Cohen’s dz. = −0.43; Competence-valuing vs. Control: *t*(33) = 1.82, *p* = 0.243, Cohen’s dz. = 0.32).

**Figure 3:**
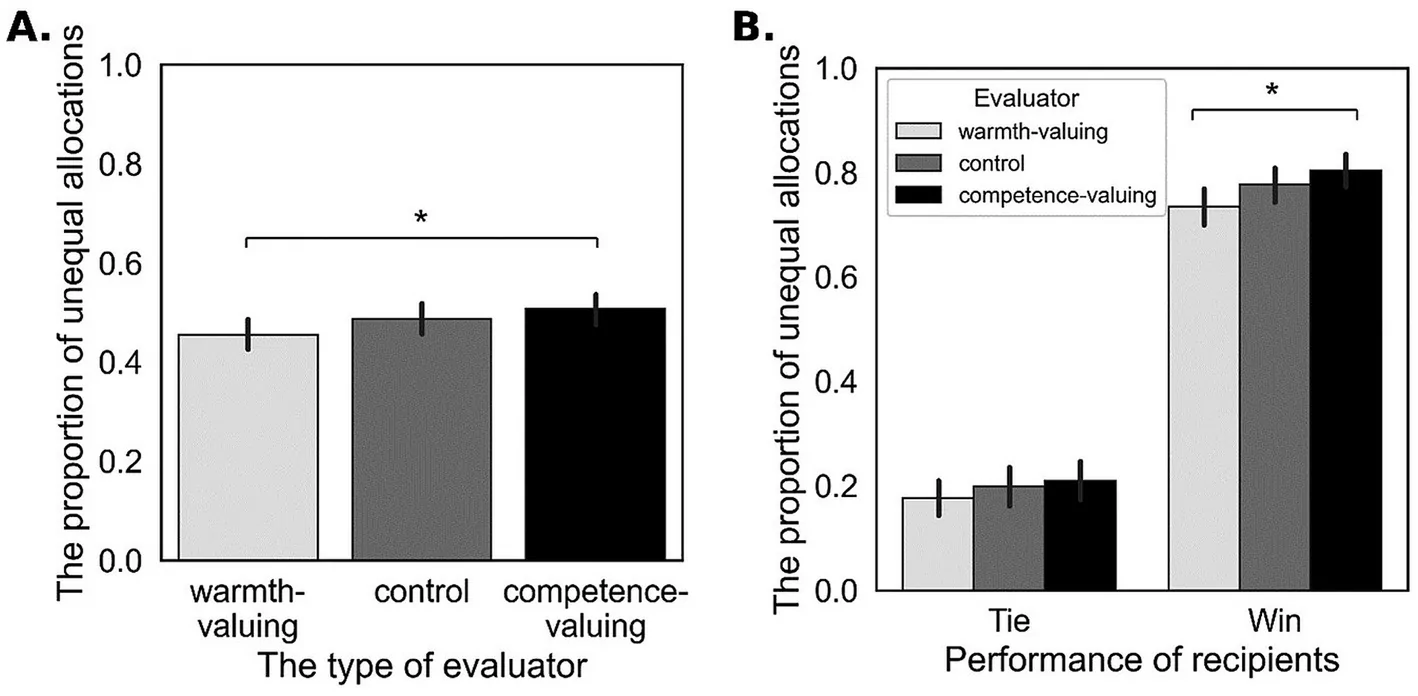
Bar plots of the choice rates averaged by **(A)** evaluator and **(B)** evaluator × performance. **(A)** Overall, participants were more likely to choose unequal allocations when observed by the competence-valuing evaluator compared to the warmth-valuing evaluator. **(B)** In Experiment 2, this pattern emerged only in the ‘Win’ condition: Participants preferred strict equality when observed by the warmth-valuing evaluator, but they preferred merit-based allocation when observed by the competence-valuing evaluator. Error bars indicate the 95% confidence interval. The asterisk indicates the significance level as follows: * (*p* < 0.05).

To clarify the overall main effect of evaluator type, we conducted follow-up analyses by each performance condition. The evaluator effect was significant only in the ‘Win’ condition (*F*(2, 32) = 3.61, *p* = 0.038, partial *η*^2^ = 0.18, 95% CI = [0.00, 0.40]), not in the ‘Tie’ condition [*F*(2, 32) = 1.47, *p* = 0.25, partial *η*^2^ = 0.08]. In the ‘Win’ condition, participants preferred merit-based (unequal) allocation under competence-valuing evaluator compared to the warmth-valuing evaluator (Competence-valuing vs. Warmth-valuing: *t*(33) = 2.74, *p* = 0.030, Cohen’s dz. = 0.48, 95% CI = [0.11, 0.83]; Warmth-valuing vs. Control: *t*(33) = −2.37, *p* = 0.072, Cohen’s dz. = −0.41; Competence-valuing vs. Control: *t*(33) = 2.09, *p* = 0.134, Cohen’s dz. = 0.36; Figure 3B).

We also observed significant main effects of performance (*F*(1, 33) = 129.43, *p* < 0.001, partial *η*^2^ = 0.80, 95% CI = [0.66, 0.87]) and ΔEfficiency level (*F*(4, 30) = 14.24, *p* < 0.001, partial *η*^2^ = 0.66, 95% CI = [0.40, 0.77]). Participants favored unequal distributions more in the ‘Win’ condition, and when unequal allocations benefited recipients more. Accordingly, the performance × ΔEfficiency interaction was significant (*F*(4, 30) = 13.90, *p* < 0.001, partial *η*^2^ = 0.65, 95% CI = [0.39, 0.77]). No other significant interaction effects were found. Full statistical results of choice data are provided in Supplementary Tables S6, S7, and S8.

#### 3.2.2 RT data

The standardized RT data were analyzed using a 2 (Performance) × 3 (Evaluator Type) × 5 (ΔEfficiency Level) RM-ANOVA. Significant main effects were found for performance (*F*(1, 33) = 5.74, *p* = 0.022, partial *η*^2^ = 0.15, 95% CI = [0.00, 0.37]) and ΔEfficiency level (*F*(4, 30) = 3.70, *p* = 0.014, partial *η*^2^ = 0.33, 95% CI = [0.02, 0.52]), indicating that participants took longer to decide in the ‘Win’ condition and that decision time varied by ΔEfficiency level. Additionally, a significant interaction between performance and ΔEfficiency was observed (*F*(4, 30) = 13.25, *p* < 0.001, partial *η*^2^ = 0.64, 95% CI = [0.38, 0.76]), such that participants responded faster when the equal allocation was more efficient in the ‘Tie’ condition, but slower when the unequal allocation was more efficient in the ‘Win’ condition.

Although the main effect of evaluator type was only marginally significant [*F*(2, 32) = 3.14, *p* = 0.06, partial *η*^2^ = 0.16], further analysis revealed that the effect of evaluator type was significant only in the ‘Win’ condition (*F*(2, 32) = 4.17, *p* = 0.025, partial *η*^2^ = 0.21, 95% CI = [0.00, 0.42]). Similar to the results of choice data, participants responded faster under competence-valuing evaluators and slower under warmth-valuing evaluators (Competence-valuing vs. Warmth-valuing: *t*(33) = −2.84, *p* = 0.022, Cohen’s dz. = −0.49, 95% CI = [−0.85, −0.13]; Warmth-valuing vs. Control: *t*(33) = 1.56, *p* = 0.388, Cohen’s dz. = 0.27; Competence-valuing vs. Control: *t*(33) = −2.28, *p* = 0.088, Cohen’s dz. = −0.40). No such effect was observed in the ‘Tie’ condition [*F*(2, 32) = 1.46, *p* = 0.247, partial *η*^2^ = 0.08]. No other interaction effect was found. Full statistical results of RT data are provided in Supplementary Tables S9, S10.

#### 3.2.3 Individual differences in the extent of impression management

To investigate the personality traits related to individual differences in impression management behavior, the same analysis as in Experiment 1 was conducted. Our findings revealed that individual FNE scores were positively correlated with the degree of individual impression management under the warmth-valuing evaluator [*r*(32) = 0.40, *p* = 0.02; Figure 4A]. While the correlation between FNE score and impression management in the competence-valuing evaluator condition was not statistically significant [*r*(32) = 0.32, *p* = 0.065], a marginal positive trend was observed, similar to that in the warmth-valuing evaluator condition.

**Figure 4:**
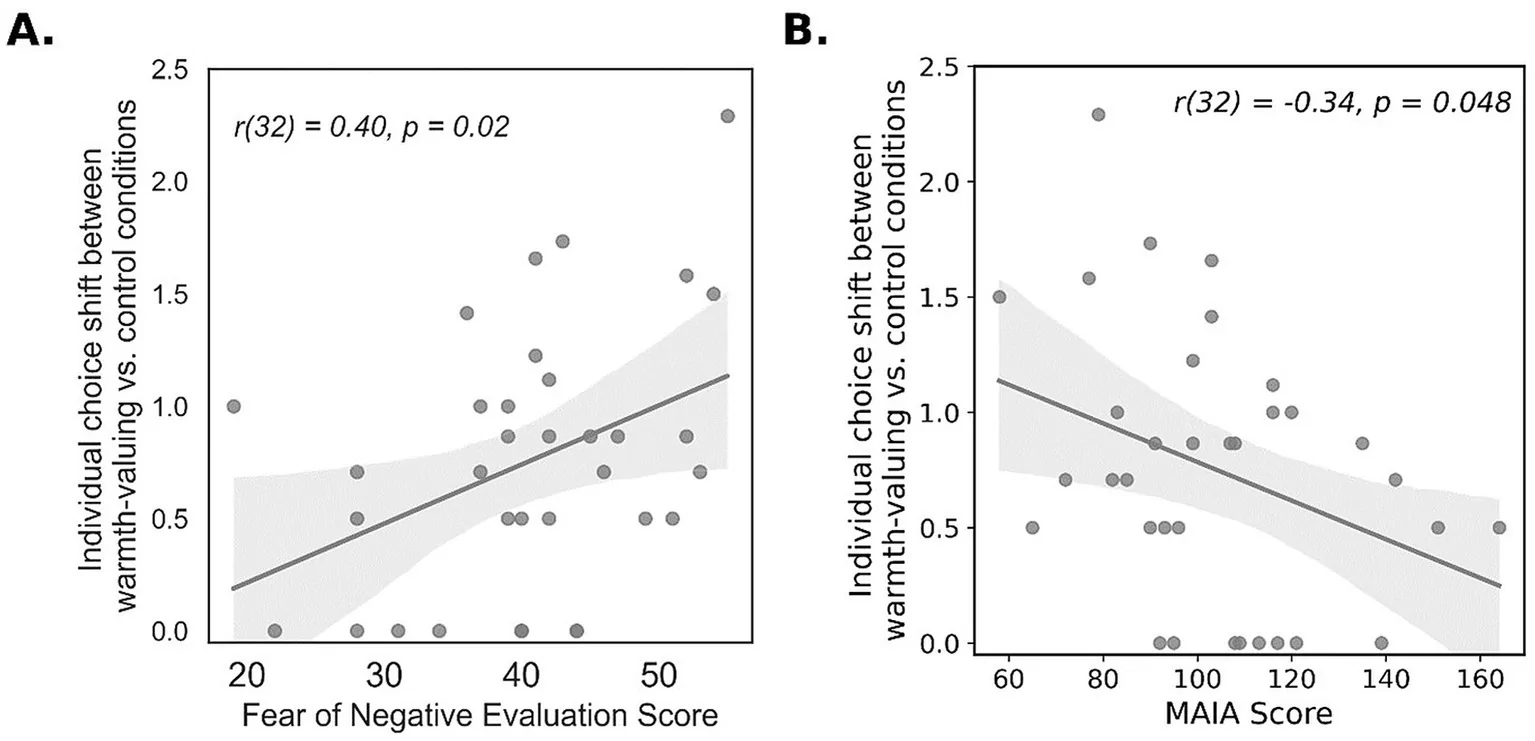
Scatter plots showing the relationship between individual impression management under the warmth-valuing evaluator and individual scores on **(A)** FNE and **(B)** K-MAIA. In Experiment 2, choice variability under the warmth-valuing evaluator was positively correlated with participants’ FNE scores. Conversely, this variability was negatively correlated with participants’ K-MAIA scores. To quantify individual variations in social influence on allocation, we calculated the absolute difference in choice patterns between the warmth-valuing condition and the control condition. The shaded areas indicate the 95% confidence interval.

Additionally, subjective interoceptive sensitivity, as measured by the K-MAIA score, tended to be negatively correlated with the degree of impression management when observed by a warmth-valuing evaluator [*r*(32) = −0.34, *p* = 0.048; competence-valuing evaluator: *r*(32) = −0.29, *p* = 0.10; Figure 4B]. To determine whether these relationships could be evident when monetary incentives were eliminated, we also applied the same correlational analyses to data from Experiment 1. However, the relationships were not observed in the Experiment 1 data. Full correlation results are provided in Supplementary Table S11.

### 3.3 Discussion

Experiment 2 showed an attenuated pattern of allocation choices compared with Experiment 1. Although participants descriptively chose unequal allocations more frequently under the competence-valuing evaluator than under the warmth-valuing evaluator, the critical interaction effects were not statistically significant, and neither evaluator condition differed significantly from the control condition. Follow-up RT analyses similarly showed a difference between the competence-valuing and warmth-valuing evaluator conditions in the ‘Win’ condition, but neither condition differed significantly from the control condition. Taken together, Experiment 2 provides only preliminary, directional evidence that evaluator orientation may be associated with variation in allocation behavior after evaluator-linked monetary incentives were removed. Because the critical interaction effects were not statistically significant and neither evaluator condition differed significantly from the control condition, Experiment 2 should not be regarded as a replication of Experiment 1, and the data do not permit firm conclusions about the independent contribution of social impression-management motives to allocation preferences.

Notably, the main effect of evaluator type replicated across both experiments, providing some convergent evidence that evaluator orientation shapes allocation choices independent of the specific experimental context. However, this main effect should be interpreted cautiously: post-hoc comparisons indicated that neither the warmth-valuing nor the competence-valuing condition differed significantly from the control condition when considered individually, and the finer-grained performance-dependent modulation that characterized Experiment 1 (i.e., the three-way interaction) did not replicate in Experiment 2. We therefore treat the significant main effect as partial, convergent support for the broader hypothesis, rather than as a full replication of the mechanism identified in Experiment 1.

The correlations between behavioral indices related to impression management and individual trait scores collected through questionnaires were observed only in Experiment 2 and not in Experiment 1. However, given the exploratory nature of these analyses, these cross-experiment differences cannot establish that monetary incentives diluted impression-management motivation. They raise the possibility that evaluator-linked monetary incentives or other methodological differences between the experiments may moderate these associations, a possibility that requires further confirmation.

## 4 General discussion

This study provides novel evidence that individuals may shift their preferences between strict equality and merit-based equity in response to others’ social impression goals, specifically warmth and competence, a pattern that was clearly observed when evaluator selections were linked to monetary incentives (Experiment 1) and emerged as a more tentative, directional pattern when they were absent (Experiment 2). Unlike previous research, which examined impression management in one-shot or between-subjects paradigms, our within-subject design enabled a direct observation of how allocators tailor their decisions to convey desired impressions. This finding is significant because it shows that decisions about fairness are not solely driven by abstract moral principles or material outcomes, but also by the desire to shape how one is perceived by others. Participants anticipated that warmth-valuing evaluators would prefer equal distributions, while competence-valuing evaluators would favor merit-based allocations, and adjusted their behavior accordingly, an effect most clearly observed in Experiment 1, and echoed, in attenuated and non-significant form, in Experiment 2.

Consequently, the behavioral adjustments observed in Experiment 1 must reflect the combined influence of impression-management goals and instrumental reward maximization, and thus should not be interpreted as demonstrating pure impression-management processes, given that evaluators’ selections were directly linked to monetary incentives. To untangle these confounded motives, Experiment 2 deliberately removed all financial incentives; however, the resulting behavioral pattern was notably attenuated. Therefore, the findings from Experiment 2 should be taken as preliminary, exploratory evidence that requires further confirmation, rather than as a robust replication of Experiment 1.

We designed a novel task assessing how individuals adapt allocation decisions to create specific social impressions, distinct from previous research using between-subjects designs or one-shot tasks. This allowed quantifying an individual propensity for impression management while distinguishing the allocator’s role from the recipient’s, exploring how the desire to project a particular social impression independently influenced allocation aspects beyond prosocial tendencies. Participants seemed to intuitively grasp which allocations would project desired impressions (warmth or competence), even without explicit information about evaluators’ preferences. This behavior persisted even when monetary incentives were removed, providing preliminary, directional evidence of impression management’s influence extending beyond financial gain. Thus, impression management within distributive justice contexts may encompass defensive attempts to avoid negative evaluation (Leary and Kowalski, 1990), proactive motives to gain social approval and enhance perceived status (Bolino et al., 2016), and strategic efforts to signal warmth or competence depending on situational demands (Holoien and Fiske, 2013; Swencionis and Fiske, 2016).

The individual-difference findings should be strictly interpreted as exploratory. In Experiment 2, greater variability in allocation choices across evaluators was associated with a higher fear of negative evaluation (FNE) (Leary, 1983; Leary and Allen, 2011). Although FNE has been linked to maladaptive social-cognitive outcomes in prior work (Watson and Friend, 1970; Gilbert, 2000; Carleton et al., 2007; Hong and Cheung, 2015; Hertz et al., 2017; Will et al., 2020), these exploratory correlations must be treated with substantial caution. Specifically, the association involving subjective interoceptive sensitivity was highly sensitive to a single outlier in Experiment 1, and Experiment 2 relied on a smaller sample while utilizing a non-identical interoception measure. Consequently, these individual-difference patterns should be regarded as preliminary, hypothesis-generating associations rather than robust, established mechanisms, and they await further confirmation using high-powered, preregistered designs.

Moreover, associations between FNE and impression management (Leary, 1983; Leary and Allen, 2011) might align with impression management’s role in mitigating negative evaluations (Piccirillo et al., 2016) or prediction errors. FNE has also been linked to intolerance of uncertainty (Carleton et al., 2010) and psychological inflexibility (Uğur et al., 2021; Kırca et al., 2024). Consequently, individuals with higher FNE may be more reluctant to encounter unpredictable reactions from evaluators, leading to prediction errors and negative evaluations, feeling greater pressure to infer and adjust decisions to align with evaluators’ preferred distributive principles. Given the lack of replication across experiments, however, this interpretation should be treated as heuristic rather than conclusive.

Interestingly, a negative correlation emerged between individual interoceptive sensitivity and the degree of impression management. Although the role of interoceptive sensitivity in social interactions has yielded conflicting results (Hübner et al., 2021), multiple studies demonstrate that an individual’s ability to perceive their own bodily signals may render them less susceptible to social influences, as exemplified by impaired interoceptive sensitivity found in psychiatric disorders like social anxiety (Gerrans and Murray, 2020; Macpherson et al., 2021) or body dysmorphic disorder (Kaplan et al., 2014). Conversely, individuals with high interoceptive sensitivity appear less influenced by social pressures when making decisions (von Mohr et al., 2021, 2023). For example, individuals with low interoceptive sensitivity were less prone to engage in deceptive behaviors when their reputation was on the line, unlike those with high interoceptive sensitivity, who remained unaffected by potential damage to their reputation (Vabba et al., 2022). Similarly, our study revealed that individuals with high interoceptive sensitivity were less inclined to adjust their resource allocation decisions to conform to perceived social expectations, especially when motivated by impression management rather than monetary incentives (Experiment 2).

A recent study revealed that individuals with higher interoceptive sensitivity are more likely to make moral decisions aligned with group consensus across a wide range of moral dilemma scenarios (Kim and Kim, 2025). Future research incorporating direct measures of prediction error, physiological regulation, and real-time interoceptive processing could examine whether predictive coding or allostatic mechanisms contribute to social-evaluative allocation behavior (Constant et al., 2019; Theriault et al., 2021). These learned internal models based on group expectations could also lead to high resistance to conformity when faced with new conflicting information, as shown by this study and previous ones (von Mohr et al., 2021, 2023). Further research is needed to understand the precise relationship between the decision to conform and the degree of conflict between one’s internal models and others’ expectations, as well as the role of interoceptive sensitivity in modulating such a relationship.

Consistent with our hypotheses, our findings suggest an association between warm impressions and the preference for strictly equal allocation, and between competent impressions and a preference for merit-based allocation, an association that was strong under monetary incentives (Experiment 1) and more tentative in their absence (Experiment 2). These findings align closely with existing literature emphasizing the pivotal roles of warmth and competence in stereotype formation (Fiske, 2015; Fiske et al., 2002; Jenkins et al., 2018), which often predict attitudes toward outgroups (Cikara et al., 2010; Jenkins et al., 2018) and individuals (Azevedo et al., 2018). This supports the idea that these stereotypical perceptions can offer insights into another person’s cooperative or competitive inclinations from an evolutionary standpoint (Fiske et al., 2007; Eisenbruch and Krasnow, 2022) and can serve as valuable cues for predicting resource allocation decisions.

This study has some limitations to consider. Firstly, we fixed the level of inequality point at two points, which may not have consistently elicited psychological conflicts associated with unequal distribution, given subjective variation in fairness threshold perceptions. Secondly, a general bias toward the merit-based equity option exists in situations involving unequal contributions. While our results indicated a greater influence of the warmth-valuing evaluator over the competence-valuing evaluator, this may have been caused by a potential ceiling effect in the ‘Win’ condition, limiting the impact of the competent impression. Similarly, the minimal choice shifts observed in the ‘Tie’ condition warrant a cautious interpretation, as the unequal option may have lacked any socially defensible justification, potentially inducing a task-imposed floor effect rather than reflecting a genuine absence of social motivation (Starmans et al., 2017). Thirdly, all experiments were conducted online, and despite our efforts to emulate a laboratory setting, this approach may affect the ecological validity of our results. The absence of direct interpersonal contact could potentially diminish the significance of impression management, particularly evident in Experiment 2. Moreover, several boundary conditions regarding the generalizability of the present findings must be carefully noted. First, participants across both experiments were predominantly recruited from university communities in Seoul, South Korea, which may restrict the immediate applicability of these distributive trends to more demographically or culturally diverse populations. Second, Experiment 2 was constrained by a relatively small final analytical sample and an asymmetrical gender distribution, which warrants caution when interpreting the robustness of its social evaluation effects. Because the critical interaction effects in Experiment 2 were not statistically significant, the present findings cannot determine whether social-evaluative orientation alone influences allocation preferences in the absence of monetary incentives. This possibility should be tested further in studies with greater statistical power, more gender-balanced community samples, and more stringent designs that better isolate social-evaluative motives from other task-related influences. Finally, while our virtual laboratory paradigm offers a creative and engaging platform that effectively heightens the psychological sense of interaction, the psychological dynamics of social evaluation within a digitized environment may distinctively diverge from those operating in organic, face-to-face settings. Future research should aim to validate these impression-management mechanisms by utilizing larger, more gender-balanced communal samples and systematically contrasting online interactions with in-person social evaluation contexts.

A fourth limitation is the use of different interoceptive-awareness measures across experiments (MAIA-2 in Experiment 1 and K-MAIA in Experiment 2). Consequently, any cross-experiment differences in the findings related to interoceptive sensitivity may be confounded by subtle differences in the psychometric properties of the two scales, rather than solely reflecting the removal of monetary incentives. Fifth, our employment of evaluators who prioritized either warmth or competence served as a simplified proof-of-concept design intended to isolate the underlying mechanism. In ecological settings, however, observers are more likely to weigh these dimensions simultaneously, applying a more nuanced balance to their evaluations of distributive decisions.

Sixth, we did not directly assess participants’ explicit beliefs regarding whether specific allocation choices (e.g., equality vs. equity) would successfully project images of warmth or competence. Because we lacked direct mediation measures of expected social impressions, our self-presentational interpretation remains partly inferential. Consequently, the observed behavioral shifts could also be interpreted as strategic compliance with highly salient evaluator preferences and task cues, rather than purely internalized self-presentational motives. Although these two processes may coexist and overlap in behavioral execution, they are theoretically distinct: strategic compliance represents a reactive adjustment to external task demands, whereas genuine self-presentation entails an autonomous desire to project specific social traits (Leary and Kowalski, 1990). Future research should explicitly decouple task demands from internal presentational goals to more cleanly distinguish between simple compliance and autonomous impression management. Additionally, the specific task structure utilized in our paradigm may have introduced inherent statistical constraints, namely ceiling and floor effects, which could limit the sensitivity needed to detect subtle evaluator-related shifts. Specifically, in the ‘Win’ condition, unequal (merit-based) allocation is normatively justified by clear performance discrepancies, potentially creating a ceiling effect toward equity. Conversely, in the ‘Tie’ condition, unequal allocation lacks a performance-based justification, which likely induced a floor effect toward strict equality. These normative anchors may have masked or attenuated the variance driven by presentational concerns, particularly in the absence of financial incentives. To untangle these overlapping factors, future studies should implement a wider range of inequality levels, present more varied and nuanced allocation options, and critically incorporate a no-observer baseline condition. Such designs will be crucial to more rigorously isolate autonomous fairness preferences and efficiency concerns from tactical impression-management motives. Finally, our participants always made their allocation choices while being observed, preventing us from ascertaining whether impression management solely responds to external social cues or is also driven by internal motivations. Future research should aim to address these limitations to provide a more comprehensive understanding of impression management’s role in resource allocation based on principles of distributive justice.

In summary, our study highlights a novel social cognitive mechanism behind fairness decisions: the strategic use of distributive justice principles to manage social impressions. Participants flexibly adjusted their choices depending on which traits—warmth or competence—were valued by observers. This suggests that fairness preferences are not fixed, but can be shaped by contextually salient social goals. Furthermore, individual variability in these adjustments was linked to fear of negative evaluation and interoceptive sensitivity, suggesting that impression management may be shaped by underlying affective and bodily self-awareness processes. Overall, this study expands the understanding of distributive justice by integrating motivational and perceptual dimensions, bridging theories of fairness and impression formation.

## Data Availability

The datasets presented in this study can be found in online repositories. The names of the repository/repositories and accession number(s) can be found at: https://osf.io/xu9n6/.

## References

[ref1] Abele A. E. Wojciszke B. (2014). Communal and agentic content in social cognition: a dual perspective model. Adv. Exp. Soc. Psychol. 50, 195–255. doi: 10.1016/B978-0-12-800284-1.00004-7

[ref2] Adams J. S. (1965). Inequity in social exchange. Adv. Exp. Soc. Psychol. 2, 267–299. doi: 10.1016/S0065-2601(08)60108-2

[ref3] Azevedo R. T. Panasiti M. S. Maglio R. Aglioti S. M. (2018). Perceived warmth and competence of others shape voluntary deceptive behaviour in a morally relevant setting. Br. J. Psychol. 109, 25–44. doi: 10.1111/bjop.12245 28276063

[ref4] Bach P. Schenke K. C. (2017). Predictive social perception: towards a unifying framework from action observation to person knowledge. Soc. Personal. Psychol. Compass 11:e12312. doi: 10.1111/SPC3.12312

[ref5] Barrett L. F. Simmons W. K. (2015). Interoceptive predictions in the brain. Nat. Rev. Neurosci. 16, 419–429. doi: 10.1038/nrn3950, 26016744 PMC4731102

[ref6] Becker J. Asbrock F. (2012). What triggers helping versus harming of ambivalent groups? Effects of the relative salience of warmth versus competence. J. Exp. Soc. Psychol. 48, 19–27. doi: 10.1016/j.jesp.2011.06.015

[ref7] Bolino M. Long D. Turnley W. (2016). Impression management in organizations: critical questions, answers, and areas for future research. Annu. Rev. Organ. Psychol. Organ. Behav. 3, 377–406. doi: 10.1146/annurev-orgpsych-041015-062337

[ref8] Bolino M. C. Turnley W. H. (2003). More than one way to make an impression: exploring profiles of impression management. J. Manage. 29, 141–160. doi: 10.1016/S0149-2063(02)00212-X

[ref9] Caleo S. (2018). When distributive justice and gender stereotypes coincide: reactions to equity and equality violations. J. Appl. Soc. Psychol. 48, 257–268. doi: 10.1111/JASP.12510

[ref10] Cappelen A. W. Eichele T. Hugdahl K. Specht K. Sørensen E. O. Tungodden B. (2014). Equity theory and fair inequality: a neuroeconomic study. Proc. Natl. Acad. Sci. USA 111, 15368–15372. doi: 10.1073/PNAS.1414602111, 25313056 PMC4217432

[ref11] Carleton R. N. Collimore K. C. Asmundson G. J. G. (2007). Social anxiety and fear of negative evaluation: construct validity of the BFNE-II. J. Anxiety Disord. 21, 131–141. doi: 10.1016/J.JANXDIS.2006.03.010 16675196

[ref12] Carleton R. N. Collimore K. C. Asmundson G. J. G. (2010). “It’s not just the judgements-it’s that I don’t know”: intolerance of uncertainty as a predictor of social anxiety. J. Anxiety Disord. 24, 189–195. doi: 10.1016/j.janxdis.2009.10.007 19931391

[ref13] Chae J. Kim K. Kim Y. Lim G. Kim D. Kim H. (2022). Ingroup favoritism overrides fairness when resources are limited. Sci. Rep. 12, 1–11. doi: 10.1038/s41598-022-08460-1, 35296722 PMC8927613

[ref14] Cialdini R. B. Goldstein N. J. (2004). Social influence: compliance and conformity. Annu. Rev. Psychol. 55, 591–621. doi: 10.1146/annurev.psych.55.090902.142015, 14744228

[ref15] Cikara M. Farnsworth R. A. Harris L. T. Fiske S. T. (2010). On the wrong side of the trolley track: neural correlates of relative social valuation. Soc. Cogn. Affect. Neurosci. 5, 404–413. doi: 10.1093/scan/nsq011, 20150342 PMC2999760

[ref16] Constant A. Ramstead M. J. D. Veissière S. P. L. Friston K. (2019). Regimes of expectations: an active inference model of social conformity and human decision making. Front. Psychol. 10:679. doi: 10.3389/FPSYG.2019.00679, 30988668 PMC6452780

[ref17] Cook K. S. Hegtvedt K. A. (1983). Distributive justice, equity, and equality. Annu. Rev. Sociol. 9, 217–241. doi: 10.1146/ANNUREV.SO.09.080183.001245

[ref18] Curtis S. R. Carre J. R. Mueller S. M. Jones D. N. (2023). Hiding your dark side: anticipatory impression management of communal traits. Curr. Psychol. 42, 18720–18730. doi: 10.1007/S12144-022-03039-5

[ref19] DeScioli P. Massenkoff M. Shaw A. Petersen M. B. Kurzban R. (2014). Equity or equality? Moral judgments follow the money. Proc. R. Soc. B Biol. Sci. 281:20142112. doi: 10.1098/rspb.2014.2112, 25355480 PMC4240999

[ref20] Deutsch M. (1975). Equity, equality, and need: what determines which value will be used as the basis of distributive justice? J. Soc. Issues 31, 137–149. doi: 10.1111/j.1540-4560.1975.tb01000.x

[ref21] Durante F. Fiske S. T. (2017). How social-class stereotypes maintain inequality. Curr. Opin. Psychol. 18, 43–48. doi: 10.1016/J.COPSYC.2017.07.033, 29221511 PMC6020691

[ref22] Durante F. Fiske S. T. Kervyn N. Cuddy A. J. C. Akande A. D. Adetoun B. E. . (2013). Nations’ income inequality predicts ambivalence in stereotype content: how societies mind the gap. Br. J. Soc. Psychol. 52, 726–746. doi: 10.1111/BJSO.12005, 23039178 PMC3855559

[ref23] Eisenbruch A. B. Krasnow M. M. (2022). Why warmth matters more than competence: a new evolutionary approach. Perspect. Psychol. Sci. 17, 1604–1623. doi: 10.1177/17456916211071087, 35748187

[ref24] Elenbaas L. (2019). Against unfairness: young children’s judgments about merit, equity, and equality. J. Exp. Child Psychol. 186, 73–82. doi: 10.1016/J.JECP.2019.05.009, 31203108

[ref25] Evans A. M. Krueger J. I. (2016). Bounded prospection in dilemmas of trust and reciprocity. Rev. Gen. Psychol. 20, 17–28. doi: 10.1037/gpr0000063

[ref26] Evans A. M. Rand D. G. (2019). Cooperation and decision time. Curr. Opin. Psychol. 26, 67–71. doi: 10.1016/j.copsyc.2018.05.007 29879640

[ref27] Evans A. M. van de Calseyde P. P. F. M. (2018). The reputational consequences of generalized trust. Personal. Soc. Psychol. Bull. 44, 492–507. doi: 10.1177/0146167217742886, 29251247 PMC5843024

[ref28] FeldmanHall O. Shenhav A. (2019). Resolving uncertainty in a social world. Nat. Hum. Behav. 3, 426–435. doi: 10.1038/s41562-019-0590-x, 31011164 PMC6594393

[ref29] Fiske S. T. (2015). Intergroup biases: a focus on stereotype content. Curr. Opin. Behav. Sci. 3, 45–50. doi: 10.1016/j.cobeha.2015.01.010, 27453920 PMC4955357

[ref30] Fiske S. T. (2018). Stereotype content: warmth and competence endure. Curr. Dir. Psychol. Sci. 27, 67–73. doi: 10.1177/0963721417738825, 29755213 PMC5945217

[ref31] Fiske S. T. Bai X. (2020). Vertical and horizontal inequality are status and power differences: applications to stereotyping by competence and warmth. Curr. Opin. Psychol. 33, 216–221. doi: 10.1016/j.copsyc.2019.09.014, 31747639

[ref32] Fiske S. T. Cuddy A. J. C. Glick P. (2007). Universal dimensions of social cognition: warmth and competence. Trends Cogn. Sci. 11, 77–83. doi: 10.1016/j.tics.2006.11.005 17188552

[ref33] Fiske S. T. Cuddy A. J. C. Glick P. Xu J. (2002). A model of (often mixed) stereotype content: competence and warmth respectively follow from perceived status and competition. J. Pers. Soc. Psychol. 82, 878–902. doi: 10.1037/0022-3514.82.6.878, 12051578

[ref34] Gerrans P. Murray R. J. (2020). Interoceptive active inference and self-representation in social anxiety disorder (SAD): exploring the neurocognitive traits of the SAD self. Neurosci. Conscious. 2020:niaa026. doi: 10.1093/NC/NIAA026, 39015778 PMC11249956

[ref35] Gilbert P. (2000). The relationship of shame, social anxiety and depression: the role of the evaluation of social rank. Clin. Psychol. Psychother. 7, 174–189. doi: 10.1002/1099-0879

[ref36] Gim W.-S. Sim K.-L. Cho O.-K. (2016). Korean multidimensional assessment of interoceptive awareness (K-MAIA): development and validation. Korean J. Stress Res. 24, 177–192. doi: 10.17547/KJSR.2016.24.3.177

[ref37] Gordon-Hecker T. Rosensaft-Eshel D. Pittarello A. Shalvi S. Bereby-Meyer Y. (2017). Not taking responsibility: equity trumps efficiency in allocation decisions. J. Exp. Psychol. Gen. 146, 771–775. doi: 10.1037/xge0000273, 28277692

[ref38] Goudarzi S. Badaan V. Knowles E. D. (2022). Neoliberalism and the ideological construction of equity beliefs. Perspect. Psychol. Sci. 17, 1431–1451. doi: 10.1177/17456916211053311 35536556

[ref39] Graham J. Haidt J. Nosek B. A. (2009). Liberals and conservatives rely on different sets of moral foundations. J. Pers. Soc. Psychol. 96, 1029–1046. doi: 10.1037/a0015141 19379034

[ref40] Haidt J. (2012). The Righteous Mind: Why Good People Are Divided by Politics and Religion. New York, NY: Vintage Books.

[ref41] Hertz U. Palminteri S. Brunetti S. Olesen C. Frith C. D. Bahrami B. (2017). Neural computations underpinning the strategic management of influence in advice giving. Nat. Commun. 8, 1–12. doi: 10.1038/s41467-017-02314-5 PMC5736665 29259152

[ref42] Holoien D. S. Fiske S. T. (2013). Downplaying positive impressions: compensation between warmth and competence in impression management. J. Exp. Soc. Psychol. 49, 33–41. doi: 10.1016/j.jesp.2012.09.001, 24550571 PMC3925755

[ref43] Hong R. Y. Cheung M. W. L. (2015). The structure of cognitive vulnerabilities to depression and anxiety: evidence for a common core etiologic process based on a meta-analytic review. Clin. Psychol. Sci. 3, 892–912. doi: 10.1177/2167702614553789

[ref44] Hsu M. Anen C. Quartz S. R. (2008). The right and the good: distributive justice and neural encoding of equity and efficiency. Science 320, 1092–1095. doi: 10.1126/SCIENCE.1153651 18467558

[ref45] Hübner A. M. Trempler I. Gietmann C. Schubotz R. I. (2021). Interoceptive sensibility predicts the ability to infer others’ emotional states. PLoS One 16:e0258089. doi: 10.1371/JOURNAL.PONE.0258089, 34613976 PMC8494315

[ref46] Huppert E. Cowell J. M. Cheng Y. Contreras-Ibáñez C. Gomez-Sicard N. Gonzalez-Gadea M. L. . (2019). The development of children’s preferences for equality and equity across 13 individualistic and collectivist cultures. Dev. Sci. 22:e12729. doi: 10.1111/desc.12729, 30207638

[ref47] Izuma K. (2012). The social neuroscience of reputation. Neurosci. Res. 72, 283–288. doi: 10.1016/j.neures.2012.01.003 22285602

[ref48] Izuma K. Saito D. N. Sadato N. (2010). The roles of the medial prefrontal cortex and striatum in reputation processing. Soc. Neurosci. 5, 133–147. doi: 10.1080/17470910903202559, 19705330

[ref49] Jenkins A. C. Karashchuk P. Zhu L. Hsu M. (2018). Predicting human behavior toward members of different social groups. Proc. Natl. Acad. Sci. USA 115, 9696–9701. doi: 10.1073/pnas.1719452115 PMC6166817 30201708

[ref50] Jordan J. J. Hoffman M. Bloom P. Rand D. G. (2016). Third-party punishment as a costly signal of trustworthiness. Nature 530, 473–476. doi: 10.1038/nature16981 26911783

[ref51] Jung D. Sul S. Lee M. Kim H. (2018). Social observation increases functional segregation between MPFC subregions predicting prosocial consumer decisions. Sci. Rep. 8:3368. doi: 10.1038/s41598-018-21449-z, 29463816 PMC5820324

[ref52] Kahn A. Lamm H. Nelson R. E. (1977). Preferences for an equal or equitable allocator. J. Pers. Soc. Psychol. 35, 837–844. doi: 10.1037/0022-3514.35.11.837

[ref53] Kahn A. O’Leary V. E. Krulewitz J. E. Lamm H. (1980). Equity and equality: male and female means to a just end. Basic Appl. Soc. Psych. 1, 173–197. doi: 10.1207/s15324834basp0102_6

[ref54] Kameda T. Inukai K. Higuchi S. Ogawa A. Kim H. Matsuda T. . (2016). Rawlsian maximin rule operates as a common cognitive anchor in distributive justice and risky decisions. Proc. Natl. Acad. Sci. USA 113, 11817–11822. doi: 10.1073/PNAS.1602641113, 27688764 PMC5081577

[ref55] Kaplan R. A. Enticott P. G. Hohwy J. Castle D. J. Rossell S. L. (2014). Is body dysmorphic disorder associated with abnormal bodily self-awareness? A study using the rubber hand illusion. PLoS One 9:e99981. doi: 10.1371/journal.pone.0099981, 24925079 PMC4055788

[ref56] Kazemi A. Eek D. Gärling T. (2016). Allocation of public goods: the interplay of self-interest, fairness, and group goal. Soc. Psychol. 47, 214–222. doi: 10.1027/1864-9335/a000275

[ref57] Kazemi A. Eek D. Gärling T. (2017). Equity, equal shares or equal final outcomes? Group goal guides allocations of public goods. Front. Psychol. 8:36. doi: 10.3389/FPSYG.2017.00036, 28179890 PMC5263145

[ref58] Kim J. Y. Kim H. (2025). Neural processes linking interoception to moral preferences aligned with group consensus. J. Neurosci. 45:e1114242025. doi: 10.1523/JNEUROSCI.1114-24.2025, 40324876 PMC12139586

[ref59] Kim D. Kim J. Kim H. (2021). Increased conformity to social hierarchy under public eyes. Front. Psychol. 12:636801. doi: 10.3389/fpsyg.2021.636801, 34335358 PMC8319240

[ref60] Kırca B. Saruhan V. Aydoğdu B. N. Avcu A. (2024). Mediation effect of cognitive flexibility between fear of negative evaluation and interaction anxiety. Curr. Psychol. 43, 2327–2336. doi: 10.1007/S12144-023-04495-3

[ref61] Ko S. J. Judd C. M. Stapel D. A. (2009). Stereotyping based on voice in the presence of individuating information: vocal femininity affects perceived competence but not warmth. Personal. Soc. Psychol. Bull. 35, 198–211. doi: 10.1177/0146167208326477 19141624

[ref62] Kobayashi K. Kable J. W. Hsu M. Jenkins A. C. (2022). Neural representations of others’ traits predict social decisions. Proc. Natl. Acad. Sci. USA 119:e2116944119. doi: 10.1073/PNAS.2116944119, 35605117 PMC9295729

[ref63] Konow J. Saijo T. Akai K. (2020). Equity versus equality: spectators, stakeholders and groups. J. Econ. Psychol. 77:102171. doi: 10.1016/J.JOEP.2019.05.001

[ref64] Kurzban R. DeScioli P. O’Brien E. (2007). Audience effects on moralistic punishment. Evol. Hum. Behav. 28, 75–84. doi: 10.1016/J.EVOLHUMBEHAV.2006.06.001

[ref65] Leary M. R. (1983). A brief version of the fear of negative evaluation scale. Personal. Soc. Psychol. Bull. 9, 371–375. doi: 10.1177/0146167283093007

[ref66] Leary M. R. Allen A. B. (2011). Personality and persona: personality processes in self-presentation. J. Pers. 79, 1191–1218. doi: 10.1111/j.1467-6494.2010.00704.x, 21204836

[ref67] Leary M. R. Kowalski R. M. (1990). Impression management: a literature review and two-component model. Psychol. Bull. 107, 34–47. doi: 10.1037/0033-2909.107.1.34

[ref68] Lee M. Sul S. Kim H. (2014). The impact of moral decision style on impression formation. Korean J. Soc. Pers. Psychol. 28, 201–223. doi: 10.21193/kjspp.2014.28.2.011

[ref69] Leventhal G. S. Michaels J. W. (1971). Locus of cause and equity motivation as determinants of reward allocation. J. Pers. Soc. Psychol. 17, 229–235. doi: 10.1037/h0030602

[ref70] Leventhal G. S. Michaels J. W. Sanford C. (1972). Inequity and interpersonal conflict: reward allocation and secrecy about reward as methods of preventing conflict. J. Pers. Soc. Psychol. 23, 88–102. doi: 10.1037/h0032877 5043007

[ref71] Lim G. Kim H. (2022). Distinctive roles of mPFC subregions in forming impressions and guiding social interaction based on others’ social behaviour. Soc. Cogn. Affect. Neurosci. 17, 1118–1130. doi: 10.1093/scan/nsac037, 35579251 PMC9714428

[ref72] Lindholm T. Yzerbyt V. (2018). When being nice or being smart could bring you down: compensatory dynamics in strategic self-presentation. Int. Rev. Soc. Psychol. 31, 1–11. doi: 10.5334/IRSP.136

[ref73] Macpherson M. C. Bîrlӑdeanu A. Miles L. K. (2021). Examining the relationship between subclinical levels of social anxiety and the rubber hand illusion. Acta Psychol. 212:103209. doi: 10.1016/J.ACTPSY.2020.103209 33197642

[ref74] Mehling W. E. Acree M. Stewart A. Silas J. Jones A. (2018). The multidimensional assessment of interoceptive awareness, version 2 (MAIA-2). PLoS One 13:e0208034. doi: 10.1371/JOURNAL.PONE.0208034, 30513087 PMC6279042

[ref75] Murphy R. O. Ackermann K. A. Handgraaf M. J. J. (2011). Measuring social value orientation. Judgm. Decis. Mak. 6, 771–781. doi: 10.2139/ssrn.1804189

[ref76] Oldmeadow J. A. Fiske S. T. (2010). Social status and the pursuit of positive social identity: systematic domains of intergroup differentiation and discrimination for high- and low-status groups. Group Process. Intergroup Relat. 13, 425–444. doi: 10.1177/1368430209355650, 24319344 PMC3852744

[ref77] Peterburs J. Voegler R. Liepelt R. Schulze A. Wilhelm S. Ocklenburg S. . (2017). Processing of fair and unfair offers in the ultimatum game under social observation. Sci. Rep. 7:44062. doi: 10.1038/srep44062, 28276510 PMC5343487

[ref78] Pezzulo G. Rigoli F. Friston K. (2015). Active inference, homeostatic regulation and adaptive behavioural control. Prog. Neurobiol. 134, 17–35. doi: 10.1016/J.PNEUROBIO.2015.09.001, 26365173 PMC4779150

[ref79] Piccirillo M. L. Taylor Dryman M. Heimberg R. G. (2016). Safety behaviors in adults with social anxiety: review and future directions. Behav. Ther. 47, 675–687. doi: 10.1016/J.BETH.2015.11.005 27816080

[ref80] Rom S. C. Conway P. (2018). The strategic moral self: self-presentation shapes moral dilemma judgments. J. Exp. Soc. Psychol. 74, 24–37. doi: 10.1016/J.JESP.2017.08.003

[ref81] Rom S. C. Weiss A. Conway P. (2017). Judging those who judge: perceivers infer the roles of affect and cognition underpinning others’ moral dilemma responses. J. Exp. Soc. Psychol. 69, 44–58. doi: 10.1016/j.jesp.2016.09.007

[ref82] Rosenberg S. Nelson C. Vivekananthan P. S. (1968). A multidimensional approach to the structure of personality impressions. J. Pers. Soc. Psychol. 9, 283–294. doi: 10.1037/h0026086, 5670821

[ref83] Rudert S. C. Reutner L. Greifeneder R. Walker M. (2017). Faced with exclusion: perceived facial warmth and competence influence moral judgments of social exclusion. J. Exp. Soc. Psychol. 68, 101–112. doi: 10.1016/J.JESP.2016.06.005

[ref84] Russell A. M. T. Fiske S. T. (2008). It’s all relative: competition and status drive interpersonal perception. Eur. J. Soc. Psychol. 38, 1193–1201. doi: 10.1002/ejsp.539

[ref85] Schäfer M. Haun D. B. M. Tomasello M. (2015). Fair is not fair everywhere. Psychol. Sci. 26, 1252–1260. doi: 10.1177/0956797615586188 26115962

[ref86] Shapiro E. G. (1975). Effect of expectations of future interaction on reward allocations in dyads: equity or equality. J. Pers. Soc. Psychol. 31, 873–880. doi: 10.1037/h0076703

[ref87] Simon J. Styczynski N. Gutsell J. (2020). Social perceptions of warmth and competence influence behavioral intentions and neural processing. Cogn. Affect. Behav. Neurosci. 20, 265–275. doi: 10.3758/S13415-019-00767-3, 31965474 PMC7220095

[ref88] Starmans C. Sheskin M. Bloom P. (2017). Why people prefer unequal societies. Nat. Hum. Behav. 1:0082. doi: 10.1038/s41562-017-0082

[ref89] Stevens C. Kristof A. (1995). Making the right impression: a field study of applicant impression management during job interviews. J. Appl. Psychol. 80, 587–606. doi: 10.1037/0021-9010.80.5.587

[ref90] Swencionis J. K. Fiske S. T. (2016). Promote up, ingratiate down: status comparisons drive warmth-competence tradeoffs in impression management. J. Exp. Soc. Psychol. 64, 27–34. doi: 10.1016/j.jesp.2016.01.004

[ref91] Tamir D. I. Thornton M. A. (2018). Modeling the predictive social mind. Trends Cogn. Sci. 22, 201–212. doi: 10.1016/j.tics.2017.12.005, 29361382 PMC5828990

[ref92] Theriault J. E. Young L. Barrett L. F. (2021). The sense of should: a biologically-based framework for modeling social pressure. Phys Life Rev 36, 100–136. doi: 10.1016/J.PLREV.2020.01.004, 32008953 PMC8645214

[ref93] Thornton M. A. Weaverdyck M. E. Tamir D. I. (2019). The brain represents people as the mental states they habitually experience. Nat. Commun. 10:2291. doi: 10.1038/s41467-019-10309-7, 31123269 PMC6533269

[ref94] Turnley W. Bolino M. (2001). Achieving desired images while avoiding undesired images: exploring the role of self-monitoring in impression management. J. Appl. Psychol. 86, 351–360. doi: 10.1037/0021-9010.86.2.351 11393446

[ref95] Uğur E. Kaya Ç. Tanhan A. (2021). Psychological inflexibility mediates the relationship between fear of negative evaluation and psychological vulnerability. Curr. Psychol. 40, 4265–4277. doi: 10.1007/S12144-020-01074-8, 32982124 PMC7509823

[ref96] Vabba A. Porciello G. Panasiti M. S. Aglioti S. M. (2022). Interoceptive influences on the production of self-serving lies in reputation risk conditions. Int. J. Psychophysiol. 177, 34–42. doi: 10.1016/J.IJPSYCHO.2022.04.001 35413427

[ref97] Veissière S. P. L. Constant A. Ramstead M. J. D. Friston K. J. Kirmayer L. J. (2020). Thinking through other minds: a variational approach to cognition and culture. Behav. Brain Sci. 43:e90. doi: 10.1017/S0140525X19001213, 31142395

[ref98] von Mohr M. Finotti G. Esposito G. Bahrami B. Tsakiris M. (2023). Social interoception: perceiving events during cardiac afferent activity makes people more suggestible to other people’s influence. Cognition 238:105502. doi: 10.1016/J.COGNITION.2023.105502 37336022

[ref99] von Mohr M. Finotti G. Villani V. Tsakiris M. (2021). Taking the pulse of social cognition: cardiac afferent activity and interoceptive accuracy modulate emotional egocentricity bias. Cortex 145, 327–340. doi: 10.1016/j.cortex.2021.10.004 34794068

[ref100] Watson D. Friend R. (1970). Measurement of social-evaluative anxiety. J. Consult. Clin. Psychol. 35:69. doi: 10.1037/h0020196 5810590

[ref101] Wei Z. Zhao Z. Zheng Y. (2013). Neural mechanisms underlying social conformity in an ultimatum game. Front. Hum. Neurosci. 7:896. doi: 10.3389/fnhum.2013.00896, 24399954 PMC3872043

[ref102] Weiss B. Feldman R. S. (2006). Looking good and lying to do it: deception as an impression management strategy in job interviews. J. Appl. Soc. Psychol. 36, 1070–1086. doi: 10.1111/J.0021-9029.2006.00055.X

[ref103] Will G. J. Moutoussis M. Womack P. M. Bullmore E. T. Goodyer I. M. Fonagy P. . (2020). Neurocomputational mechanisms underpinning aberrant social learning in young adults with low self-esteem. Transl. Psychiatry 10:96. doi: 10.1038/s41398-020-0702-4, 32184384 PMC7078312

[ref104] Yoon L. Kim K. Jung D. Kim H. (2021). Roles of the MPFC and insula in impression management under social observation. Soc. Cogn. Affect. Neurosci. 16, 474–483. doi: 10.1093/scan/nsab008, 33449108 PMC8095000

[ref105] Zhao K. Ferguson E. Smillie L. D. (2017). When fair is not equal: compassion and politeness predict allocations of wealth under different norms of equity and need. Soc. Psychol. Personal. Sci. 8, 847–857. doi: 10.1177/1948550616683018

