# Supplementary material for: Impression management in distributive justice: balancing fairness and social perception
Source: Front Psychol. 2026 Sep 18;17:1848060. doi: 10.3389/fpsyg.2026.1848060 (PMC13631039; doi:10.3389/fpsyg.2026.1848060)
Supplement: Supplementary file 1 [file Data_Sheet_1.pdf]

Supplementary Material

1 Supplementary Text 1. Experimental stimuli and tasks

1.1 Settings and stimuli

We conducted our study in a virtual lab environment created via Gather (https://www.gather.town), accessible to participants through their personal laptops or desktops. This platform allowed for the construction of an interactive virtual map, where participants and experimenters were represented by avatars. Each experimental session involved five avatars: two participants, an experimenter, and two confederates. Participants and confederates could interact with each other through text chat or audio. They moved their avatars to designated task rooms, where participants could engage with tasks or surveys by pressing the 'x' key in front of a computer icon, mimicking real-life labs (see Supplementary Figure 1).

The online tasks were developed using the Cognition website (https://www.cognition.run), which was built on the jsPsych framework (version 7.2.1). This enabled the visualization of stimuli and the collection of response data directly from web browsers.

To help participants distinguish each evaluator during tasks, we used character images separate from Gather avatars. To address the potential influence of gender, we matched evaluator characters to the gender of the participants. Thus, we created images of ten male and ten female characters. The character images were generated using the mobile apps—'Unniedoll' for females and 'Oppadoll' for males (created by 'Supercent. Inc'). An independent online sample (N = 53) then rated these images based on the eight traits, categorized into warmth-related (warm, friendly, well-intentioned, good-natured) and competence-related (competent, intelligent, confident, skillful) (Fiske et al., 2002). These raters used a 5-point Likert scale (1: highly unlikely – 5: highly likely) to guess how likely each character was to have each trait word. Scores for warmth and competence-related words were averaged to create a 'warm score' and a 'competent score' for each avatar.

The final image selection was based on two criteria. First, each character had to exhibit a significant difference between its 'warm' and 'competent' scores to ensure that participants could easily distinguish between the two types of evaluators. Second, we matched the scores across genders to ensure parity. Finally, the character chosen as the 'warm evaluator' had a warm score of 4.34 (or 4.34 for the female character) and a competent score of 3.70 (or 3.51). Conversely, the 'competent evaluator' had scores of 2.61 (or 2.70) and 3.64 (or 3.77) in the warm and competent categories, respectively.

1.2 Pre-visit phase

Before accessing the online laboratory, participants were required to provide two pieces of information: 1) ratings on the importance they place on eight social traits, and 2) a personal character image. As these were suggested as each evaluator's information to manipulate experimental conditions, these were not used in the study. However, they served to convince participants that role assignments were not predetermined.

First, participants completed an online survey asking how much they value each trait for evaluating other people or themselves. The traits were the same four warmth- and competence-related traits used to select the evaluators' images. Instead, each trait was rated on a 4-point Likert scale (1: 'highly insignificant' - 4: 'highly significant').

Second, participants created an image of their own character and used the same app that created the evaluator images. They were guided to customize various facial features and costumes, aiming to create a character that represented them.

1.3 Cognitive task

In the cognitive tasks, allocators who were actual participants performed a memory task, while evaluators who were confederates performed a dot estimation task. We instructed participants that this task measures the social memory ability of allocators and the number perception ability of evaluators. The actual purpose of the cognitive task was to manipulate participants through the experimental conditions, which hinted to participants which impression they should project in each evaluator's trials.

In the memory task (Figure S2), the two allocators had to remember four traits most valued by two evaluators, information acquired in the pre-visit phase. The task began by displaying the character images of the two evaluators alongside their valuing traits. One evaluator, represented by a 'warm' character image, valued four warmth-related traits. The other, represented by a 'competent' character image, valued four competence-related traits. However, they were never explicitly described as 'warm' or 'competent' evaluators to avoid the demand characteristics.

Each trial presented one evaluator's character image with multiple trait words. Allocators had to confirm if all the listed traits correctly matched the evaluator's image. Up to four traits could be presented simultaneously. The practice session of the memory task comprised 28 true and 12 false trials, and feedback was provided. Incorrect answers were marked with a red 'X.' The trials were repeated until a participant reached 90% accuracy. All participants successfully completed the practice session. The test session consisted of 12 true and 8 false trials, and no feedback was given. All participants achieved over 80% accuracy (mean accuracy = 97%) in the test session. They received KRW 50 per correct trial as an incentive.

Simultaneously, participants were informed that evaluators were completing a dot estimation task, requiring them to quickly and accurately judge if the number of dots on the screen exceeded a given number. The quicker and more accurate evaluator would win each round, or it would be a tie if both were wrong or equally fast. This task served two functions: it prevented participants from suspicion about the purpose of the memory task and provided a cover story for the performance results of prior participants that would be suggested in the social allocation task.

2 Supplementary Figures and Tables

Figure S1. The screenshot of the online laboratory. Participants accessed the online laboratory and navigated each experimental room with their avatars. In each session, every participant and experimenter moved from one task room to another. In each room, they listened to task instructions by clicking the 'x' key on document icons and completed tasks with computer icons. During the social allocation task, avatars representing allocators sat at red desks and evaluator avatars sat at blue desks (left middle).

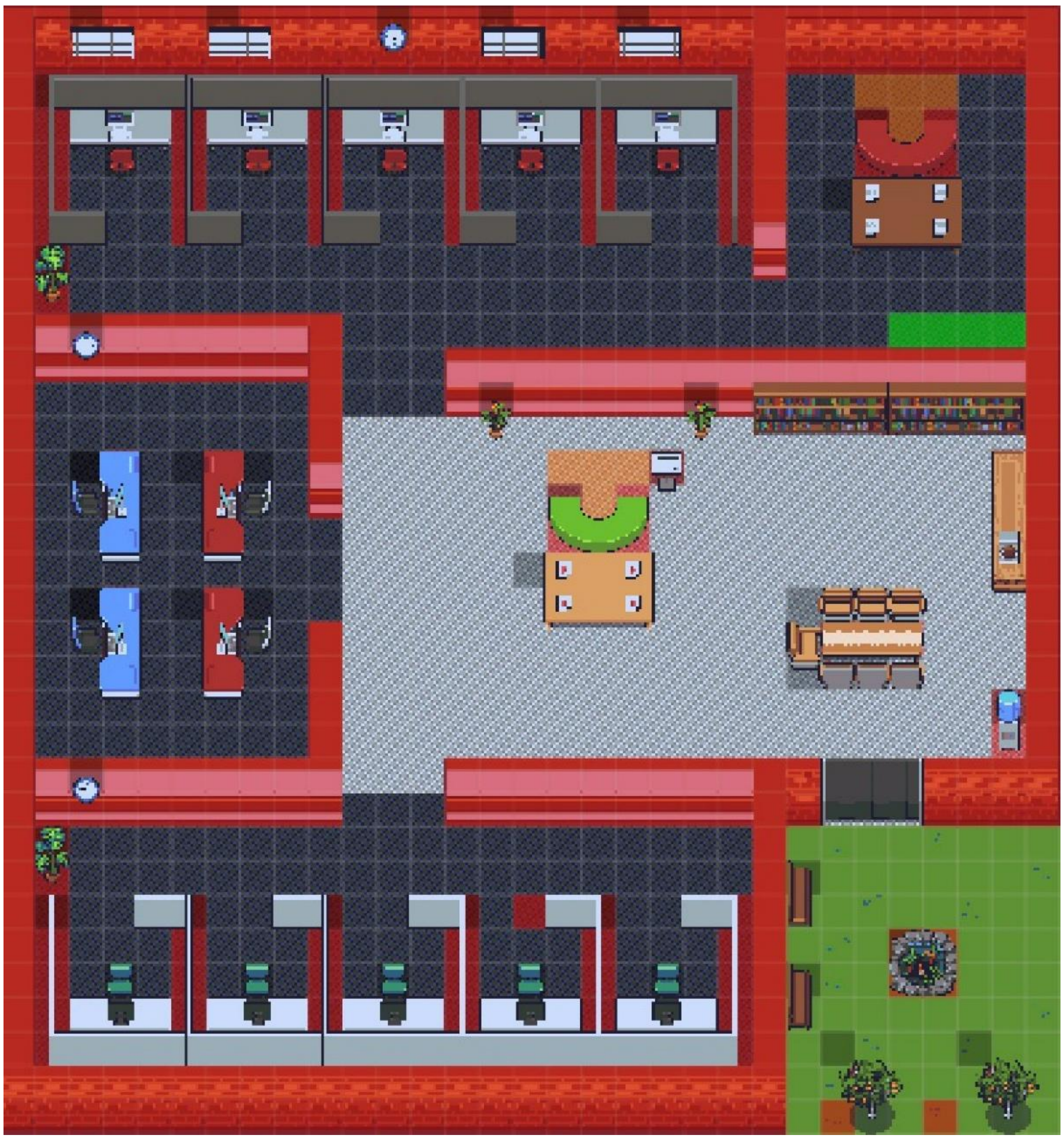

Figure S2. The example of memory task screens. (Left) Trait-encoding screen. At the start of the memory task, two evaluator character images appeared, each paired with four trait words—warmth traits on the left, competence traits on the right. Participants advanced at their own pace. **(Right)** Task trial screen. Participants had to press 'O' ('F' key) for image-word match or 'X' ('J' key) for mismatch. During the practice session, an incorrect response is followed by a red 'X' as feedback. Trials repeated until 90% accuracy.

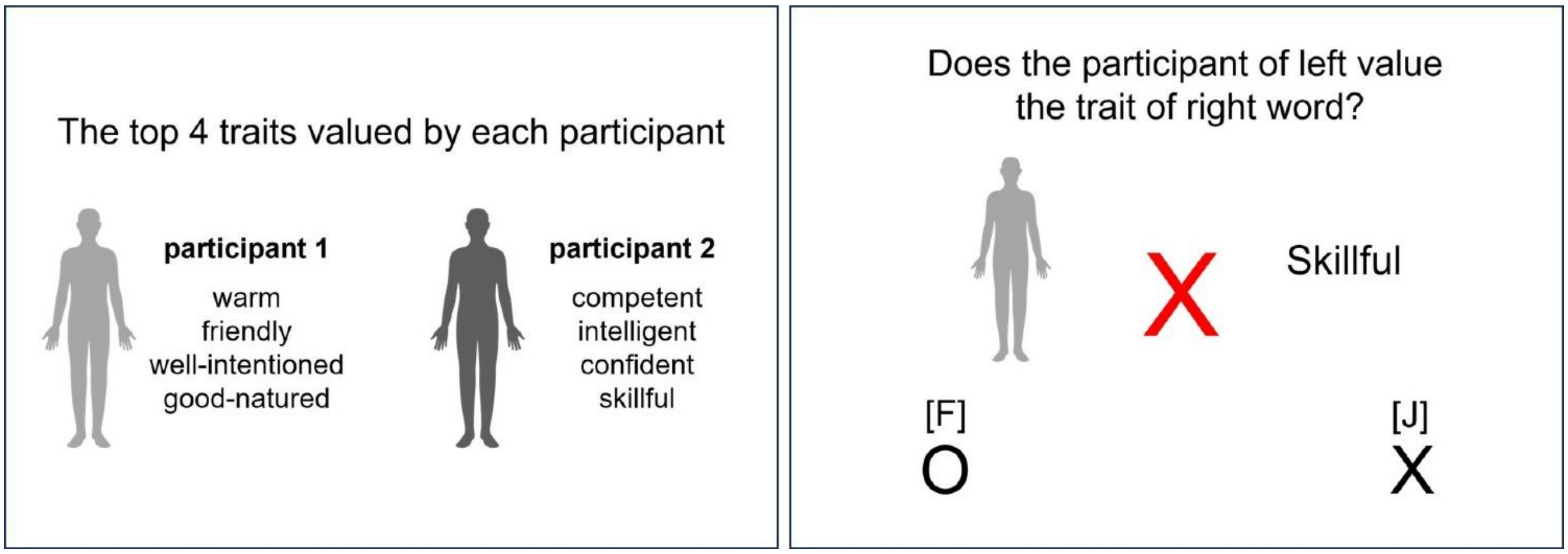

Table S1. RM-ANOVA results of 3 (Evaluator) × 2 (Performance) × 5 (ΔEfficiency) design and follow-up 3 (Evaluator) × 5 (ΔEfficiency) analyses within each performance condition on choice rates (Experiment 1)

Variable	F	Hypothesis df	Error df	p-value	Partial η^2	95% confidence interval	
						lower bound	upper bound
**A. 3 (Evaluator) × 2 (Performance) × 5 (ΔEfficiency) RM-ANOVA**							
**Evaluator**	**25.84**	**2**	**59**	**<.001**	**0.47**	**0.27**	**0.60**
**Performance**	**148.44**	**1**	**60**	**<.001**	**0.71**	**0.59**	**0.79**
**ΔEfficiency**	**28.35**	**4**	**57**	**<.001**	**0.67**	**0.51**	**0.76**
**Evaluator × Performance**	**15.53**	**2**	**59**	**<.001**	**0.34**	**0.15**	**0.50**
Evaluator × ΔEfficiency	1.61	8	53	0.143	0.20	0.00	0.31
**Performance × ΔEfficiency**	**13.97**	**4**	**57**	**<.001**	**0.49**	**0.29**	**0.62**
**Evaluator × Performance * ΔEfficiency**	**2.57**	**8**	**53**	**0.019**	**0.28**	**0.01**	**0.40**
**B. Tie Condition Only: 3 (Evaluator) × 5 (ΔEfficiency) RM-ANOVA**							
Evaluator	2.89	2	59	0.064	0.09	0.00	0.23

**ΔEfficiency**	**19.01**	**4**	**57**	**<.001**	**0.57**	0.38	0.68
Evaluator × ΔEfficiency	0.58	8	53	0.789	0.08	0.00	0.12
**C. Win Condition Only: 3 (Evaluator) × 5 (ΔEfficiency) RM-ANOVA**							
**Evaluator**	**27.74**	**2**	**59**	**<.001**	**0.48**	0.29	0.62
**ΔEfficiency**	**36.58**	**4**	**57**	**<.001**	**0.72**	0.58	0.80
**Evaluator × ΔEfficiency**	**3.43**	**8**	**53**	**0.003**	**0.34**	0.06	0.47

* Statistically significant effects are highlighted in bold.

Table S2. Post-hoc comparisons of choice rates by evaluator conditions (Experiment 1, Bonferroni-corrected)

A. Main effect of Evaluator (Evaluator contrasts collapsed across conditions)

Evaluator Contrast	Mean Difference	Std. Error	p-value	t-value	cohen's dz	95% confidence interval	
						lower bound	upper bound
**Competence-valuing > Warmth-valuing**	**0.14**	**0.02**	**<.001**	**7.16**	**0.92**	**0.62**	**1.22**
**Competence-valuing > Control**	**0.04**	**0.01**	**<.001**	**3.89**	**0.50**	**0.23**	**0.77**
**Control > Warmth-valuing**	**0.10**	**0.02**	**<.001**	**6.31**	**0.81**	**0.52**	**1.10**

B. Evaluator × Performance interaction (Evaluator contrasts effects at each Performance level)

Performance	Evaluator Contrast	Mean Difference	Std. Error	p-value	t-value	cohen's dz	95% confidence interval	
							lower bound	upper bound
Tie	Competence-valuing > Warmth-valuing	0.04	0.02	0.11	2.17	0.28	0.02	0.54
	Competence-valuing > Control	0.00	0.02	1	-0.24	-0.03	-0.28	0.22
	Control > Warmth-valuing	0.04	0.02	0.092	2.20	0.28	0.02	0.54
Win	**Competence-valuing > Warmth-valuing**	**0.23**	**0.03**	**<.001**	**7.52**	**0.97**	**0.66**	**1.27**

		**Competence-valuing > Control**	**0.08**	**0.02**	**<.001**	**4.69**	**0.61**	**0.33**	**0.88**
		**Control > Warmth-valuing**	**0.16**	**0.03**	**<.001**	**6.08**	**0.78**	**0.49**	**1.07**

C. Evaluator × Performance × ∆Efficiency interaction (Evaluator contrasts at each Performance × ∆Efficiency level)

△Efficiency	Performance	Evaluator Condition	Mean Difference	Std. Error	p-value	t-value	cohen's dz	95% conficdence interval	
								lower bound	upper bound
-4	Tie	Competence-valuing > Warmth-valuing	0.03	0.02	0.328	1.65	0.21	-0.04	0.47
		Competence-valuing > Control	0.02	0.02	1	0.70	0.09	-0.16	0.34
		Control > Warmth-valuing	0.02	0.01	0.781	1.14	0.15	-0.11	0.40
	Win	**Competence-valuing > Warmth-valuing**	**0.24**	**0.04**	**<.001**	**5.52**	**0.71**	**0.43**	**0.99**
		**Competence-valuing > Control**	**0.09**	**0.03**	**0.01**	**3.00**	**0.39**	**0.12**	**0.65**
		**Control > Warmth-valuing**	**0.16**	**0.04**	**<.001**	**4.33**	**0.56**	**0.29**	**0.83**
-2	Tie	Competence-valuing > Warmth-valuing	0.02	0.02	0.754	1.16	0.15	-0.11	0.40
		Competence-valuing > Control	0.01	0.03	1	0.18	0.02	-0.23	0.28
		Control > Warmth-valuing	0.02	0.02	1	0.67	0.09	-0.17	0.34
	Win	**Competence-valuing > Warmth-valuing**	**0.35**	**0.05**	**<.001**	**7.65**	**0.99**	**0.68**	**1.29**

		**Competence-valuing > Control**	**0.18**	**0.04**	**<.001**	**4.73**	**0.61**	**0.33**	**0.88**
		**Control > Warmth-valuing**	**0.18**	**0.04**	**<.001**	**4.68**	**0.60**	**0.33**	**0.88**
0	Tie	Competence-valuing > Warmth-valuing	0.04	0.03	0.516	1.38	0.18	-0.08	0.43
		Competence-valuing > Control	-0.02	0.03	1	-0.81	-0.11	-0.36	0.15
		Control > Warmth-valuing	0.07	0.03	0.134	2.06	0.27	0.01	0.52
	Win	**Competence-valuing > Warmth-valuing**	**0.26**	**0.05**	**<.001**	**5.42**	**0.70**	**0.41**	**0.98**
		Competence-valuing > Control	0.06	0.03	0.071	2.33	0.30	0.04	0.56
		**Control > Warmth-valuing**	**0.20**	**0.04**	**<.001**	**5.05**	**0.65**	**0.37**	**0.93**
2	Tie	Competence-valuing > Warmth-valuing	0.04	0.04	0.964	1.00	0.13	-0.13	0.38
		Competence-valuing > Control	-0.03	0.03	1	-0.90	-0.12	-0.37	0.14
		Control > Warmth-valuing	0.07	0.04	0.222	1.82	0.24	-0.02	0.49
	Win	**Competence-valuing > Warmth-valuing**	**0.18**	**0.04**	**<.001**	**5.00**	**0.65**	**0.37**	**0.92**
		Competence-valuing > Control	0.03	0.02	0.354	1.56	0.20	-0.05	0.46
		**Control > Warmth-valuing**	**0.15**	**0.03**	**<.001**	**4.69**	**0.61**	**0.33**	**0.88**
4	Tie	Competence-valuing > Warmth-valuing	0.06	0.05	0.705	1.20	0.15	-0.10	0.41
		Competence-valuing > Control	0.01	0.04	1	0.12	0.02	-0.24	0.27

		Control > Warmth-valuing	0.05	0.04	0.709	1.20	0.15	-0.10	0.41
	Win	**Competence-valuing > Warmth-valuing**	**0.14**	**0.03**	**<.001**	**4.28**	**0.55**	**0.28**	**0.82**
		Competence-valuing > Control	0.03	0.01	0.25	1.79	0.23	-0.03	0.49
		**Control > Warmth-valuing**	**0.11**	**0.03**	**0.001**	**3.73**	**0.48**	**0.21**	**0.75**

* Statistically significant effects are highlighted in bold.

Table S3. RM-ANOVA results of 3 (Evaluator) × 2 (Performance) × 5 (ΔEfficiency) design and follow-up 3 (Evaluator) × 5 (ΔEfficiency) analyses within each performance condition on (standardized) reaction time (Experiment 1)

Variable	F	Hypothesis df	Error df	p-value	Partial η^2	95% confidence interval	
						lower bound	upper bound
**A. 3 (Evaluator) × 2 (Performance) × 5 (ΔEfficiency) RM-ANOVA**							
**Evaluator**	**5.74**	**2**	**59**	**0.005**	**0.16**	**0.02**	**0.32**
**Performance**	**30.89**	**1**	**60**	**<.001**	**0.34**	**0.16**	**0.50**
ΔEfficiency	1.82	4	57	0.137	0.11	0.00	0.24
**Evaluator × Performance**	**9.47**	**2**	**59**	**<.001**	**0.24**	**0.07**	**0.41**
**Evaluator × ΔEfficiency**	**3.37**	**8**	**53**	**0.003**	**0.34**	**0.06**	**0.47**
**Performance × ΔEfficiency**	**10.24**	**4**	**57**	**<.001**	**0.42**	**0.20**	**0.56**
Evaluator × Performance × ΔEfficiency	0.98	8	53	0.464	0.13	0.00	0.21
**B. Tie Condition Only: 3 (Evaluator) × 5 (ΔEfficiency) RM-ANOVA**							
Evaluator	0.56	2	59	0.576	0.02	0.00	0.11
**ΔEfficiency**	**6.80**	**4**	**57**	**<.001**	**0.32**	**0.11**	**0.47**

**Evaluator × ΔEfficiency**	**2.30**	**8**	**53**	**0.034**	**0.26**	**0.00**	**0.38**
**C. Win Condition Only: 3 (Evaluator) × 5 (ΔEfficiency) RM-ANOVA**							
**Evaluator**	**20.87**	**2**	**59**	**<.001**	**0.41**	**0.22**	**0.56**
**ΔEfficiency**	**14.32**	**4**	**57**	**<.001**	**0.50**	**0.29**	**0.63**
**Evaluator × ΔEfficiency**	**2.14**	**8**	**53**	**0.048**	**0.24**	**0.00**	**0.37**

* Statistically significant effects are highlighted in bold.

Table S4. Post-hoc comparisons of (standardized) reaction time by evaluator conditions (Experiment 1, Bonferroni-corrected)

**A. Main effect of Evaluator** (Evaluator contrasts collapsed across conditions)								
	Evaluator Contrast	Mean Difference	Std. Error	p-value	t-value	cohen's dz	95% confidence interval	
							lower bound	upper bound
	**Competence-valuing > Warmth-valuing**	**-0.10**	**0.03**	**0.005**	**-3.33**	**-0.43**	**-0.69**	**-0.16**
	**Competence-valuing > Control**	-0.06	0.03	0.117	-2.11	-0.27	-0.53	-0.01
	**Control > Warmth-valuing**	-0.04	0.03	0.581	-1.31	-0.17	-0.42	0.09
**B. Evaluator × Performance interaction** (Evaluator contrasts effects at each Performance level)								
Performance	Evaluator Contrast	Mean Difference	Std. Error	p-value	t-value	cohen's dz	95% confidence interval	
							lower bound	upper bound
Tie	Competence-valuing > Warmth-valuing	0.02	0.05	1	0.36	0.05	-0.21	0.30
	Competence-valuing > Control	-0.03	0.04	1	-0.70	-0.09	-0.34	0.16
	Control > Warmth-valuing	0.05	0.05	0.916	1.02	0.13	-0.12	0.39
Win	**Competence-valuing > Warmth-valuing**	**-0.22**	**0.03**	**<.001**	**-6.41**	**-0.83**	**-1.12**	**-0.53**
	**Competence-valuing > Control**	**-0.09**	**0.03**	**0.031**	**-2.67**	**-0.34**	**-0.60**	**-0.08**

	**Control > Warmth-valuing**	**-0.13**	**0.04**	**0.006**	**-3.25**	**-0.42**	**-0.68**	**-0.15**

* Statistically significant effects are highlighted in bold.

Table S5. Correlation matrix of individual variables (Experiment 1)

		MFQ Harm/Care	MFQ_ Fairness/ Reciprocity	MFQ_ Ingroup/Loyalty	MFQ_ Authority/ Respect	MFQ_ Purity/Sanctity	SVO angle	BFNE-2	MAIA-2	Self-rated Warmth	Self-rated Competence	Distance between warmth vs. control condition
MFQ_ Fairness/Reciprocity	*r*	**.396****										
	p	**0.002**										
	N	**61**										
MFQ_ Ingroup/Loyalty	*r*	0.204	0.104									
	p	0.115	0.426									
	N	61	61									
MFQ_ Authority/Respect	*r*	0.069	-0.049	**.468****								
	p	0.596	0.706	**0.000**								
	N	61	61	**61**								
MFQ_ Purity/Sanctity	*r*	**.292***	0.028	**.323***	**.366****							
	p	**0.023**	0.831	**0.011**	**0.004**							

	N	**61**	61	**61**	**61**					
SVO angle	*r*	0.127	-0.093	0.063	0.006	0.015				
	p	0.329	0.475	0.627	0.963	0.910				
	N	61	61	61	61	61				
BFNE-2	*r*	0.198	**.278***	0.136	0.177	0.200	0.019			
	p	0.126	**0.030**	0.295	0.173	0.121	0.885			
	N	61	**61**	61	61	61	61			
MAIA-2	*r*	-0.104	-0.205	0.024	0.058	0.129	-0.015	-0.087		
	p	0.425	0.114	0.851	0.658	0.323	0.907	0.507		
	N	61	61	61	61	61	61	61		
Self-rated Warmth	*r*	**.265***	0.172	**.428****	0.171	**.305***	0.032	0.189	0.160	
	p	**0.039**	0.185	**0.001**	0.188	**0.017**	0.805	0.145	0.217	
	N	**61**	61	**61**	61	**61**	61	61	61	
Self-rated Competence	*r*	-0.151	0.058	-0.154	-0.031	-0.183	-0.143	-0.059	**.289***	-0.125
	p	0.245	0.657	0.235	0.812	0.158	0.272	0.653	**0.024**	0.337
	N	61	61	61	61	61	61	61	**61**	61

Individual distance between warmth vs. control condition	*r*	0.104	-0.058	0.126	0.116	-0.024	-0.129	-0.074	-0.065	-0.033	-0.092	
	p	0.426	0.659	0.335	0.373	0.856	0.320	0.572	0.621	0.800	0.480	
	N	61	61	61	61	61	61	61	61	61	61	
Individual distance between competence vs. control condition	*r*	0.024	-0.190	-0.048	0.007	-0.127	-0.027	0.070	**-.266***	0.003	-0.088	**.275***
	p	0.851	0.142	0.714	0.955	0.329	0.837	0.593	**0.038**	0.980	0.501	**0.032**
	N	61	61	61	61	61	61	61	**61**	61	61	**61**

**: $p < 0.01$, *: $p < 0.05$

Table S6. Results of a 3 (Evaluator) × 2 (Performance) × 5 (ΔEfficiency) repeated-measures ANOVA on choice rates (Experiment 2)

Variable	F	Hypothesis df	Error df	p-value	Partial η^2	95% confidence interval	
						lower bound	upper bound
**Evaluator**	**3.74**	**2**	**32**	**0.035**	**0.19**	**0.00**	**0.40**
**Performance**	**129.43**	**1**	**33**	**<.001**	**0.80**	**0.66**	**0.87**
**ΔEfficiency**	**14.24**	**4**	**30**	**<.001**	**0.66**	**0.40**	**0.77**
Evaluator × Performance	1.20	2	32	0.314	0.07	**0.00**	**0.26**
Evaluator × ΔEfficiency	0.61	8	26	0.765	0.16	0.00	0.24
**Performance × ΔEfficiency**	**13.90**	**4**	**30**	**<.001**	**0.65**	**0.39**	**0.77**
**Evaluator × Performance × ΔEfficiency**	0.35	8	26	0.939	0.10	**0.00**	**0.09**

* Statistically significant effects are highlighted in bold.

Table S7. Post-hoc comparisons of choice rates by evaluator conditions (Study 2, Bonferroni-corrected)

Evaluator Contrast	Mean Difference	Std. Error	p-value	t-value	cohen's dz	95% confidence interval	
						lower bound	upper bound
**Competence-valuing > Warmth-valuing**	**0.05**	**0.02**	**0.027**	**2.68**	**0.47**	**0.10**	**0.82**
**Competence-valuing > Control**	0.02	0.01	0.243	1.82	0.32	-0.04	0.66
**Control > Warmth-valuing**	0.03	0.01	0.072	2.46	0.43	0.07	0.78

* Statistically significant effects are highlighted in bold.

Table S8. Evaluator × ΔEfficiency RM-ANOVA and post-hoc evaluator comparisons within each performance condition on choice data (Study 2, Bonferroni-corrected)

A. Tie Condition

Evaluator × ΔEfficiency RM-ANOVA

Variable	F	Hypothesis df	Error df	p-value	Partial η^2	95% confidence interval	
						lower bound	upper bound
Evaluator	1.47	2	32	0.245	0.08	0.00	0.28
**ΔEfficiency**	**10.30**	**4**	**30**	**0.000**	**0.58**	**0.29**	**0.72**
Evaluator × ΔEfficiency	0.58	8	26	0.783	0.15	0.00	0.23

Paired t-tests on evaluator effects

Evaluator Contrast	Mean Difference	Std. Error	p-value	t-value	cohen's dz	95% confidence interval	
						lower bound	upper bound
Competence-valuing > Warmth-valuing	0.03	0.02	0.283	1.72	0.30	-0.05	0.65
Competence-valuing > Control	0.01	0.01	0.974	1.00	0.17	-0.17	0.52
Control > Warmth-valuing	0.02	0.02	0.681	1.23	0.21	-0.13	0.56

B. Win Condition

Evaluator × ΔEfficiency RM-ANOVA

Variable	F	Hypothesis df	Error df	p-value	Partial η^2	95% confidence interval	
						lower bound	upper bound
**Evaluator**	**3.61**	**2**	**32**	**0.038**	**0.18**	**0.00**	**0.40**
**ΔEfficiency**	**13.68**	**4**	**30**	**0.000**	**0.65**	**0.39**	**0.77**
Evaluator × ΔEfficiency	1.13	8	26	0.379	0.26	0.00	0.40

Paired t-tests on evaluator effects

Evaluator Contrast	Mean Difference	Std. Error	p-value	t-value	cohen's dz	95% confidence interval	
						lower bound	upper bound
**Competence-valuing > Warmth-valuing**	**0.07**	**0.03**	**0.030**	**2.74**	**0.48**	**0.11**	**0.83**
Competence-valuing > Control	0.03	0.01	0.134	2.09	0.36	0.01	0.71
Control > Warmth-valuing	0.04	0.02	0.072	2.37	0.41	0.05	0.76

* Statistically significant effects are highlighted in bold.

Table S9. Results of a 3 (Evaluator) × 2 (Performance) × 5 (ΔEfficiency) repeated-measures ANOVA on (standardized) reaction time (Experiment 2)

Variable	F	Hypothesis df	Error df	p-value	Partial η^2	95% confidence interval	
						lower bound	upper bound
Evaluator	3.14	2	32	0.057	0.16	0.00	0.38
**Performance**	**5.74**	**1**	**33**	**0.022**	**0.15**	**0.00**	**0.37**
**ΔEfficiency**	**3.70**	**4**	**30**	**0.014**	**0.33**	0.02	0.52
Evaluator × Performance	2.33	2	32	0.113	0.13	0.00	0.33
Evaluator × ΔEfficiency	0.50	8	26	0.848	0.13	0.00	0.19
**Performance × ΔEfficiency**	**13.25**	**4**	**30**	**<.001**	**0.64**	**0.38**	**0.76**
Evaluator × Performance × ΔEfficiency	1.42	8	26	0.236	0.30	0.00	0.45

* Statistically significant effects are highlighted in bold.

Table S10. Evaluator × ΔEfficiency RM-ANOVA and post-hoc evaluator comparisons within each performance condition on (standardized) reaction time (Study 2, Bonferroni-corrected)

A. Tie Condition

Evaluator × ΔEfficiency RM-ANOVA

Variable	F	Hypothesis df	Error df	p-value	Partial η^2	95% confidence interval	
						lower bound	upper bound
Evaluator	1.46	2	32	0.247	0.08	0.00	0.28
**ΔEfficiency**	**6.47**	**4**	**30**	**0.001**	**0.46**	**0.14**	**0.63**
Evaluator × ΔEfficiency	0.59	8	26	0.774	0.15	0.00	0.23

Paired t-tests on evaluator effects

Evaluator Contrast	Mean Difference	Std. Error	p-value	t-value	cohen's dz	95% confidence interval	
						lower bound	upper bound
Competence-valuing > Warmth-valuing	0.05	0.07	1.000	0.75	0.13	-0.21	0.47
Competence-valuing > Control	-0.07	0.06	0.783	-1.14	-0.20	-0.54	0.15
Control > Warmth-valuing	0.12	0.07	0.297	1.70	0.30	-0.06	0.64

B. Win Condition

Evaluator × ΔEfficiency RM-ANOVA

Variable	F	Hypothesis df	Error df	p-value	Partial η^2	95% confidence interval	
						lower bound	upper bound
**Evaluator**	**4.17**	**2**	**32**	**0.025**	**0.21**	**0.00**	**0.42**
**ΔEfficiency**	**22.53**	**4**	**30**	**0.000**	**0.75**	**0.56**	**0.84**
Evaluator × ΔEfficiency	0.89	8	26	0.542	0.21	0.00	0.34

Paired t-tests on evaluator effects

Evaluator Contrast	Mean Difference	Std. Error	p-value	t-value	cohen's dz	95% confidence interval	
						lower bound	upper bound
**Competence-valuing > Warmth-valuing**	**-0.15**	**0.05**	**0.022**	**-2.84**	**-0.49**	**-0.85**	**-0.13**
Competence-valuing > Control	-0.09	0.04	0.088	-2.28	-0.40	-0.75	-0.04
Control > Warmth-valuing	-0.07	0.04	0.388	-1.56	-0.27	-0.62	0.08

* Statistically significant effects are highlighted in bold.

Table S11. Correlation matrix of individual variables (Experiment 2)

		MFQ_ Harm/ Care	MFQ_ Fairness/ Reciprocity	MFQ_ Ingroup/ Loyalty	MFQ_ Authority/ Respect	MFQ_ Purity/Sanctity	SVO angle	BFNE-2	K-MAIA	Self-rated Warmth	Self-rated Competence	Distance between warmth vs. control condition
MFQ_ Fairness/Reciprocity	*r*	**.400***										
	p	**0.019**										
	N	**34**										
MFQ_ Ingroup/Loyalty	*r*	0.227	0.006									
	p	0.197	0.974									
	N	34	34									
MFQ_ Authority/Respect	*r*	0.049	-0.205	**.807****								
	p	0.784	0.244	**0.000**								
	N	34	34	**34**								
MFQ_ Purity/Sanctity	*r*	0.119	-0.081	**.658****	**.663****							
	p	0.503	0.648	**0.000**	**0.000**							
	N	34	34	**34**	**34**							

SVO angle	*r*	-0.206	0.042	-0.124	-0.172	0.046					
	p	0.242	0.815	0.485	0.330	0.794					
	N	34	34	34	34	34					
BFNE-2	*r*	0.210	0.274	0.170	0.031	0.125	0.066				
	p	0.234	0.117	0.337	0.862	0.481	0.712				
	N	34	34	34	34	34	34				
K-MAIA	*r*	-0.074	0.061	0.052	-0.097	-0.015	-0.148	**-.562****			
	p	0.678	0.731	0.768	0.584	0.934	0.404	**0.001**			
	N	34	34	34	34	34	34	**34**			
Self-rated Warmth	*r*	**.506****	0.235	-0.107	-0.070	0.048	-0.097	0.148	-0.153		
	p	**0.002**	0.182	0.549	0.694	0.788	0.585	0.404	0.386		
	N	**34**	34	34	34	34	34	34	34		
Self-rated Competence	*r*	-0.054	0.185	-0.139	-0.267	-0.265	-0.161	-0.287	**.499****	0.163	
	p	0.763	0.295	0.434	0.127	0.129	0.363	0.100	**0.003**	0.356	
	N	34	34	34	34	34	34	34	**34**	34	
	r	-0.070	0.054	-0.138	0.070	-0.016	-0.101	**.396***	**-.342***	0.180	-0.112

Distance between warmth vs. control condition	*p*	0.693	0.761	0.438	0.693	0.930	0.571	**0.020**	**0.048**	0.307	0.530	
	N	34	34	34	34	34	34	**34**	**34**	34	34	
Distance between competence vs. control condition	*r*	-0.021	0.064	0.015	0.241	0.106	-0.040	0.321	-0.287	0.064	-0.050	**.818****
	p	0.906	0.717	0.931	0.170	0.552	0.822	0.065	0.100	0.719	0.779	**0.000**
	N	34	34	34	34	34	34	34	34	34	34	**34**

**: $p < 0.01$, *: $p < 0.05$
